# Supplementary material for: A haplotype-resolved chromatin landscape connects cis-regulatory variants to trait variation in Citrus
Source: BMC Genomics. 2025 Oct 30;26:978. doi: 10.1186/s12864-025-12137-0 (PMC12577126; doi:10.1186/s12864-025-12137-0)
Supplement: Supplementary file 2 — Supplementary Material 2. [file 12864_2025_12137_MOESM2_ESM.pdf]

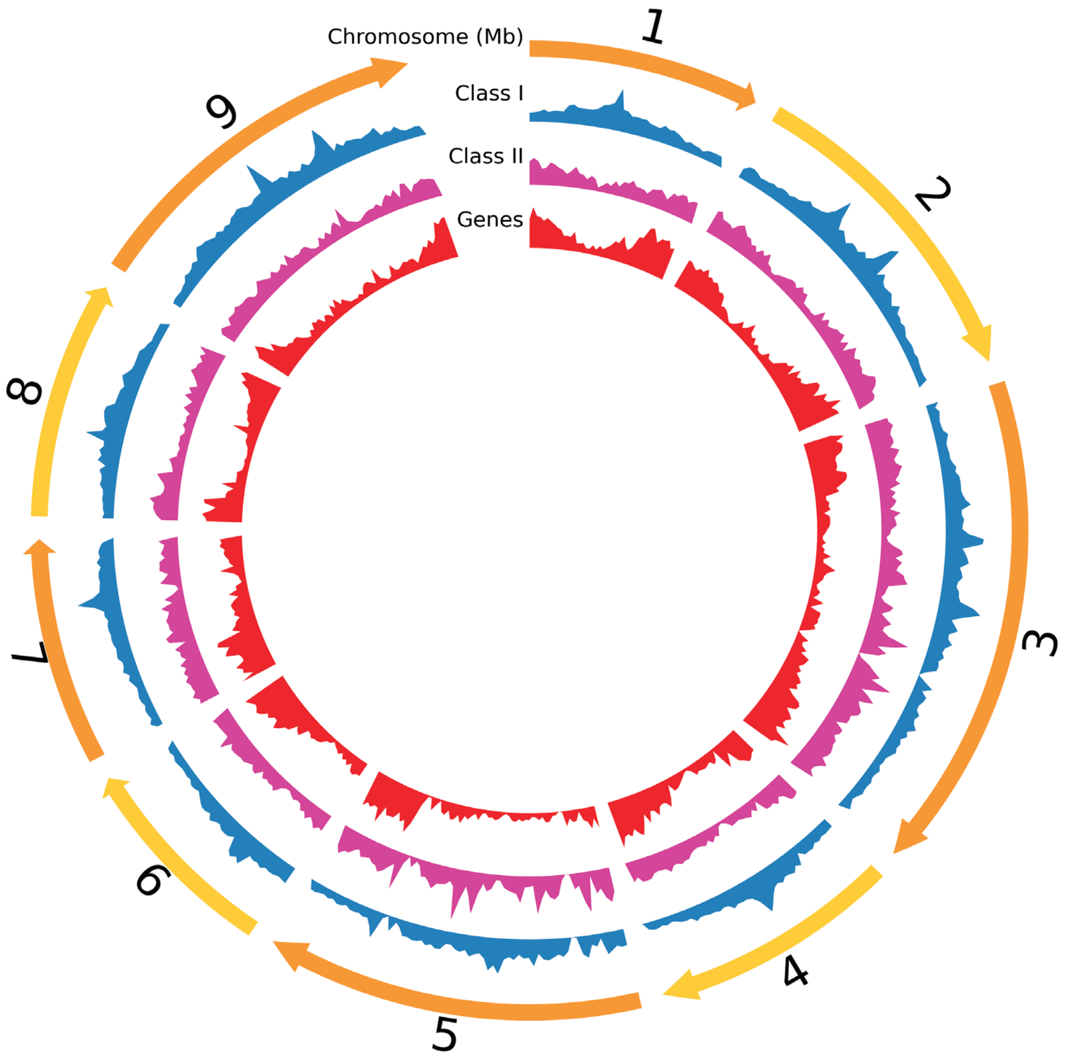

Supplemental Fig. S1. Circos plot depicting the nine chromosomes of the ‘Fairchild’ genome assembly. Chromosomes, Class I transposable elements, Class II transposable elements, and genes are indicated by separate tracks.

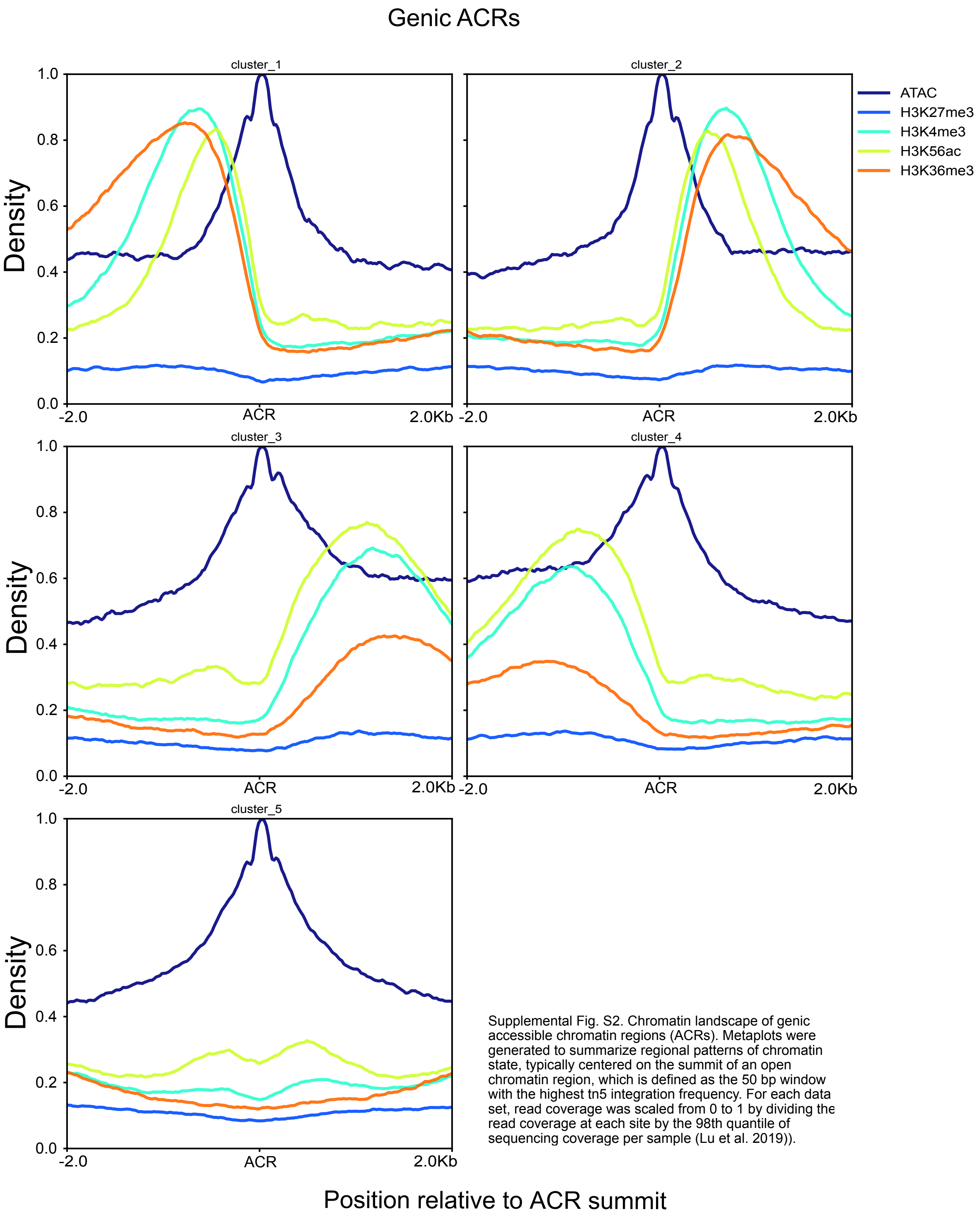

Supplemental Fig. S2. Chromatin landscape of genic accessible chromatin regions (ACRs). Metaplots were generated to summarize regional patterns of chromatin state, typically centered on the summit of an open chromatin region, which is defined as the 50 bp window with the highest tn5 integration frequency. For each data set, read coverage was scaled from 0 to 1 by dividing the read coverage at each site by the 98th quantile of sequencing coverage per sample (Lu et al. 2019)).

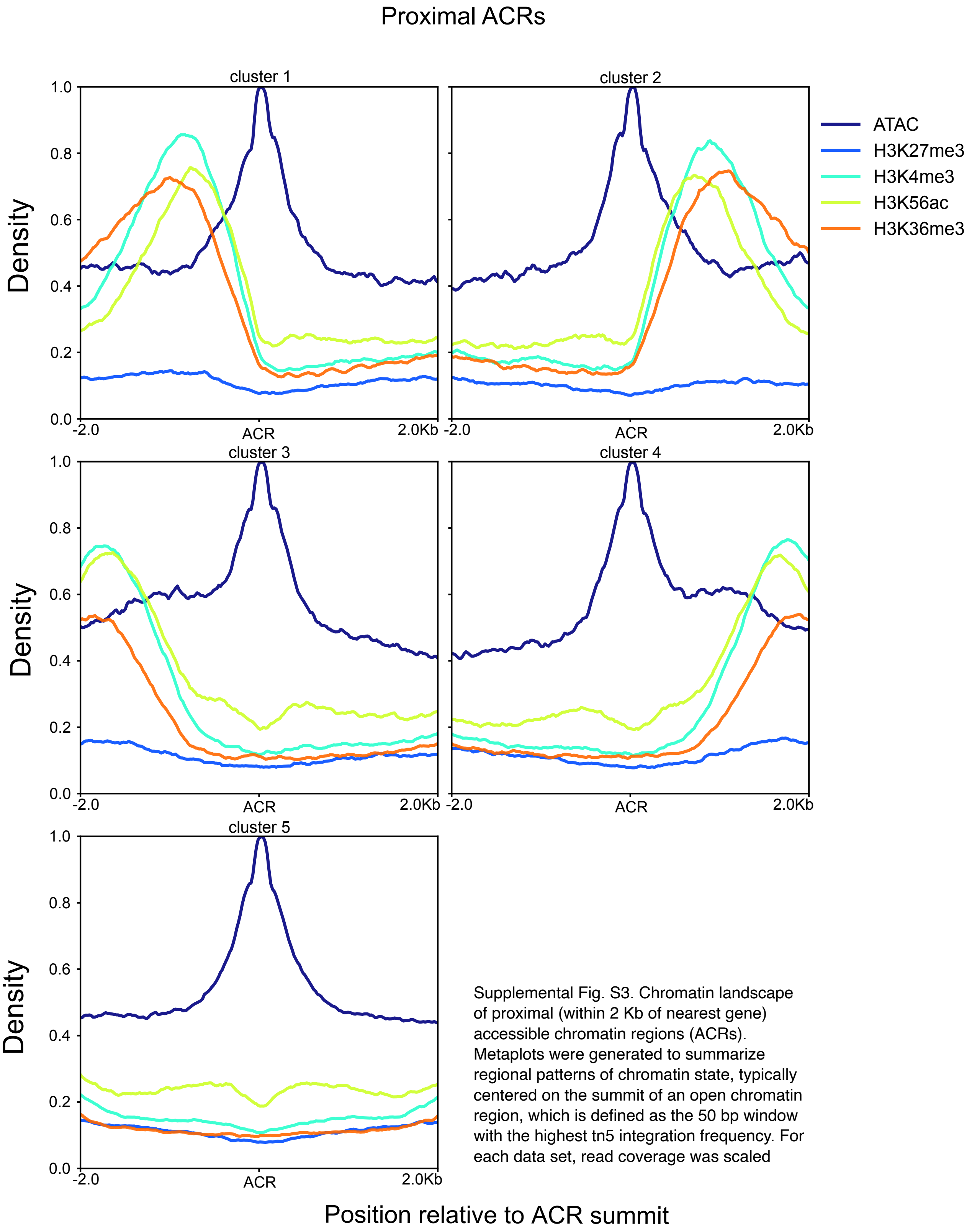

Supplemental Fig. S3. Chromatin landscape of proximal (within 2 Kb of nearest gene) accessible chromatin regions (ACRs). Metaplots were generated to summarize regional patterns of chromatin state, typically centered on the summit of an open chromatin region, which is defined as the 50 bp window with the highest tn5 integration frequency. For each data set, read coverage was scaled

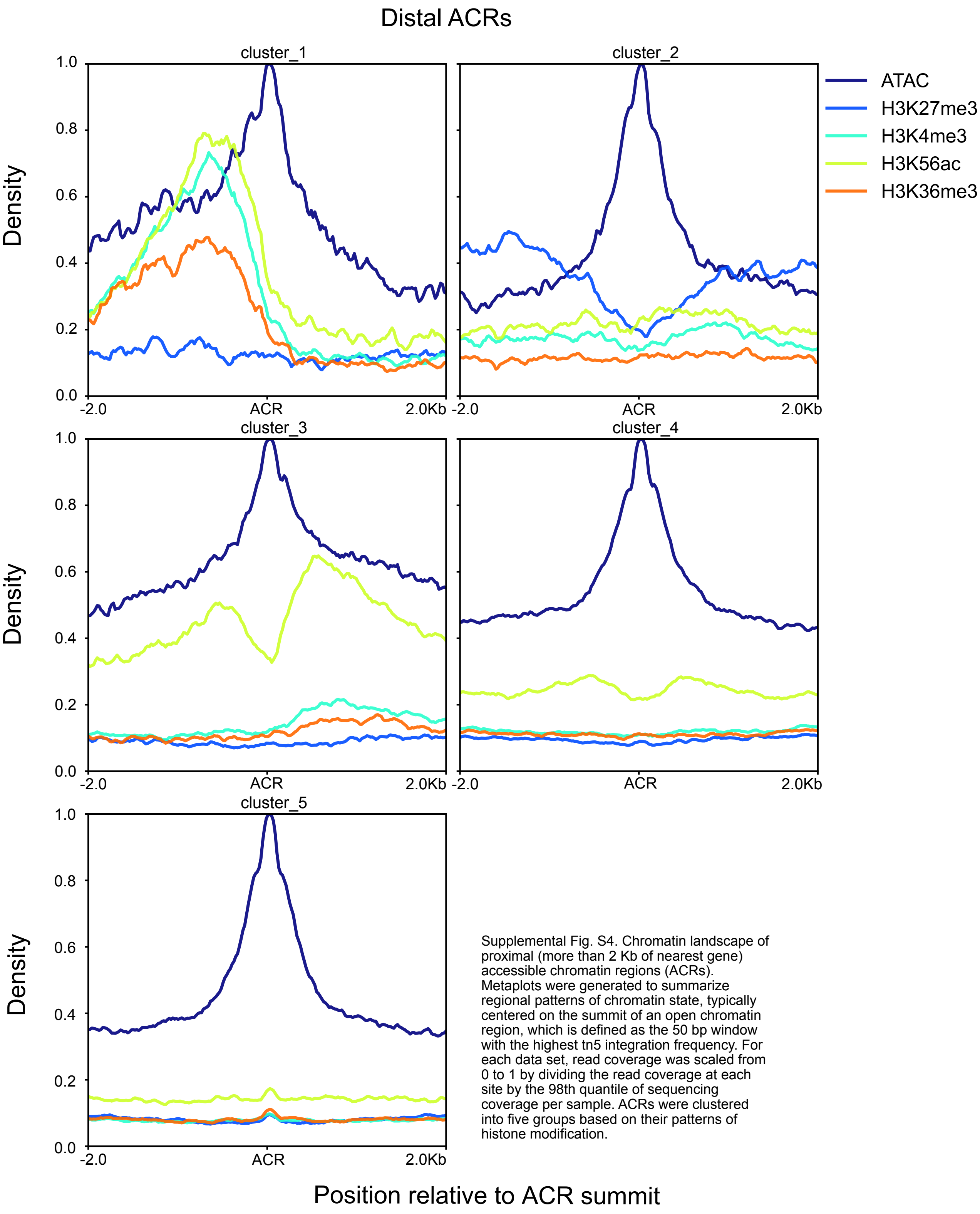

Supplemental Fig. S4. Chromatin landscape of proximal (more than 2 Kb of nearest gene) accessible chromatin regions (ACRs). Metaplots were generated to summarize regional patterns of chromatin state, typically centered on the summit of an open chromatin region, which is defined as the 50 bp window with the highest tn5 integration frequency. For each data set, read coverage was scaled from 0 to 1 by dividing the read coverage at each site by the 98th quantile of sequencing coverage per sample. ACRs were clustered into five groups based on their patterns of histone modification.

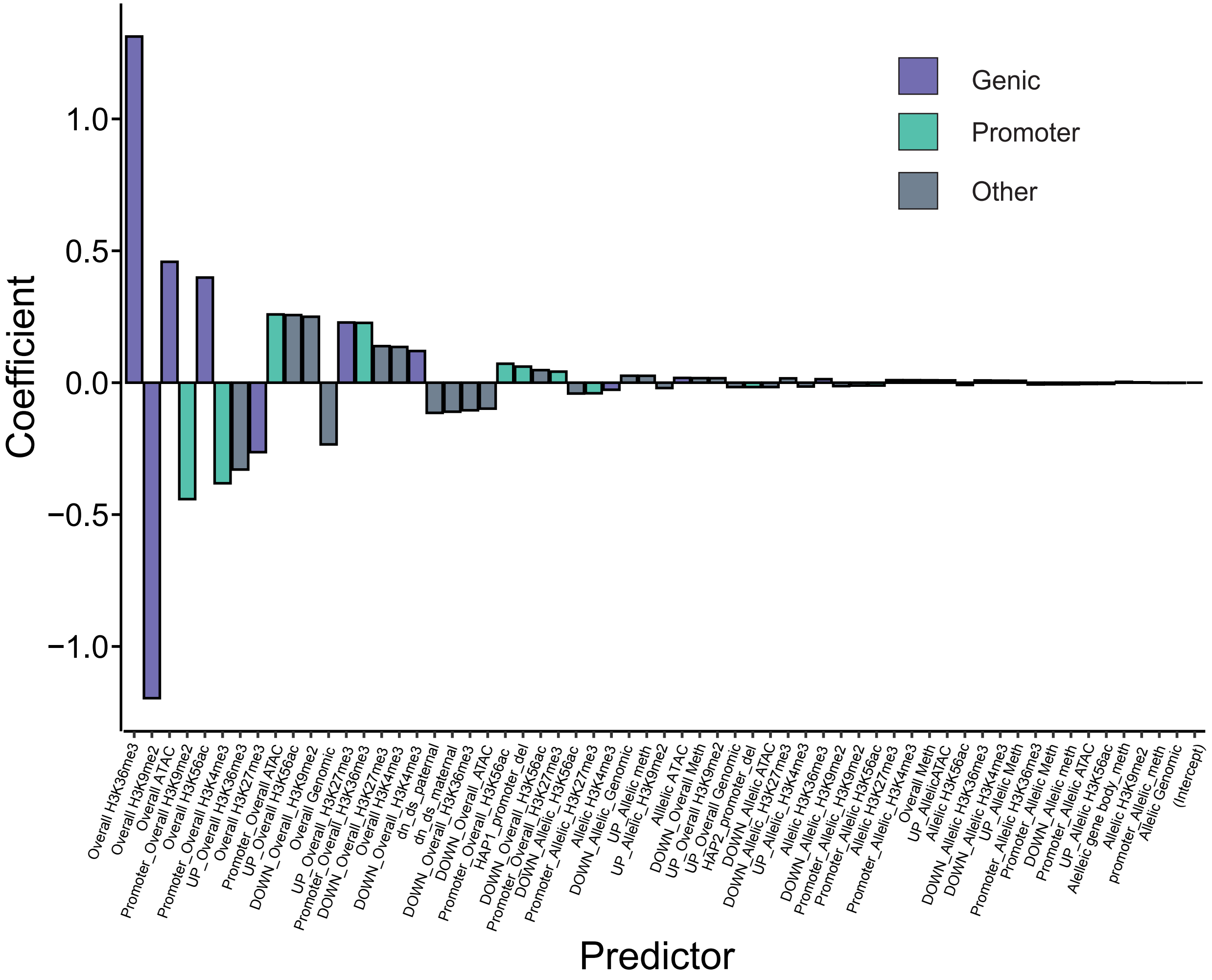

Supplemental Fig. S5. Modeling of overall gene expression of all genes in the ‘Fairchild’ genome. Coefficients for significant predictors of overall expression (log2(TPM)) of all genes (n = 30,724) ordered by magnitude (R = 65.15). Factors are partitioned by genomic region and colored to indicate whether they reside in genes, promoters (1kb of TSS), or upstream/ downstream putative regulatory regions of the focal gene (ACRs present 5 Kb upstream of promoter / 5 Kb downstream of gene). A summary of all models used is available in additional file 1: Table S12.

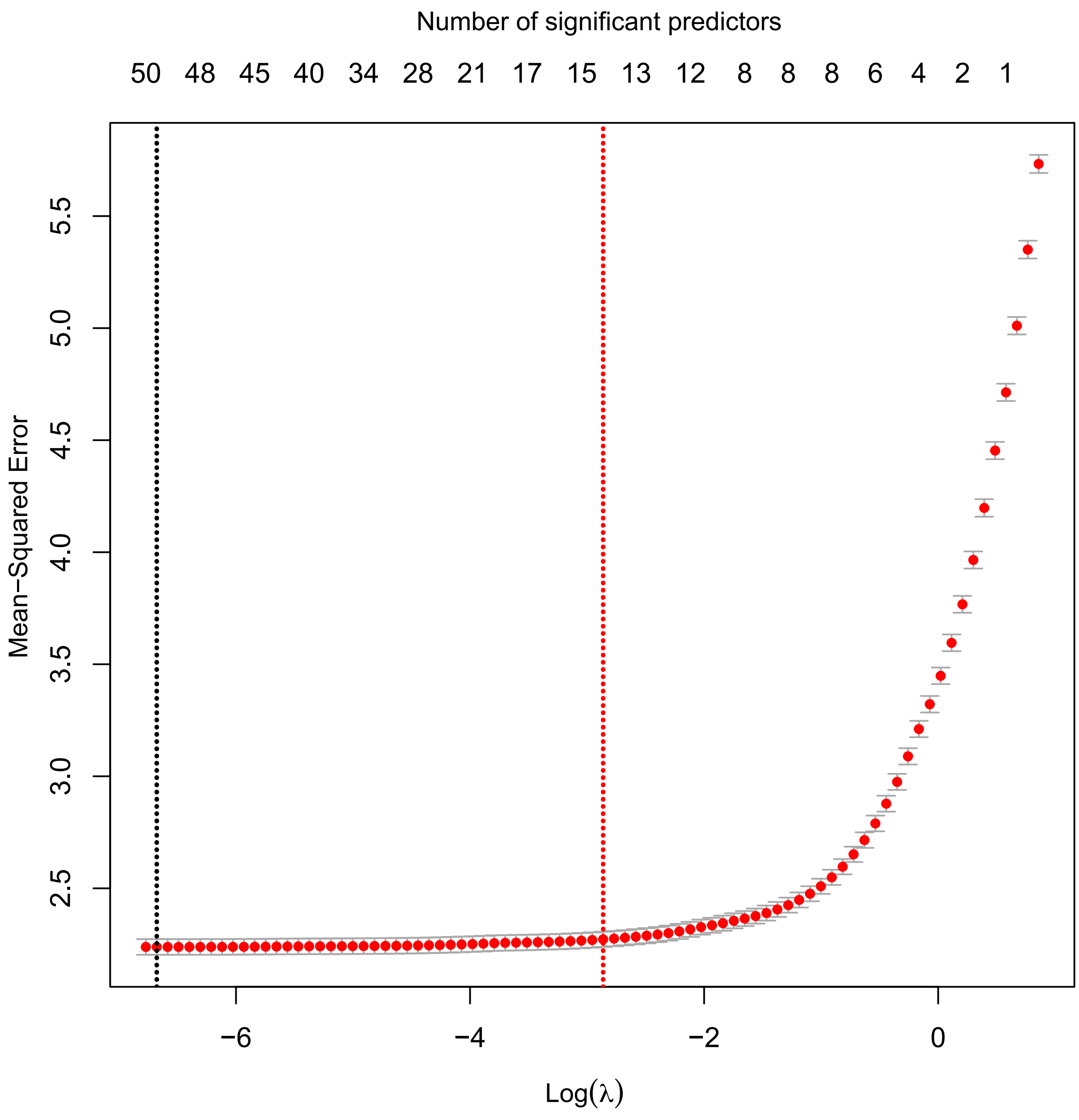

Supplemental Fig. S6. Determination of shrinkage parameter (λ) to minimize model complexity while maintaining predictive accuracy for overall expression (log2(TPM)) of all genes. The relationship between the shrinkage parameter (λ) and prediction accuracy (mean-squared error) during 5-fold cross validation with a constant tuning parameter (α = 0.70853). The dotted vertical lines represent the range of λ values that maintain a prediction accuracy within one standard-error of the model chosen through the initial cross-validation. The red vertical line indicates the λ value chosen to minimize model complexity while maintaining performance. A summary of all models used is available in additional file 1: Table S12.

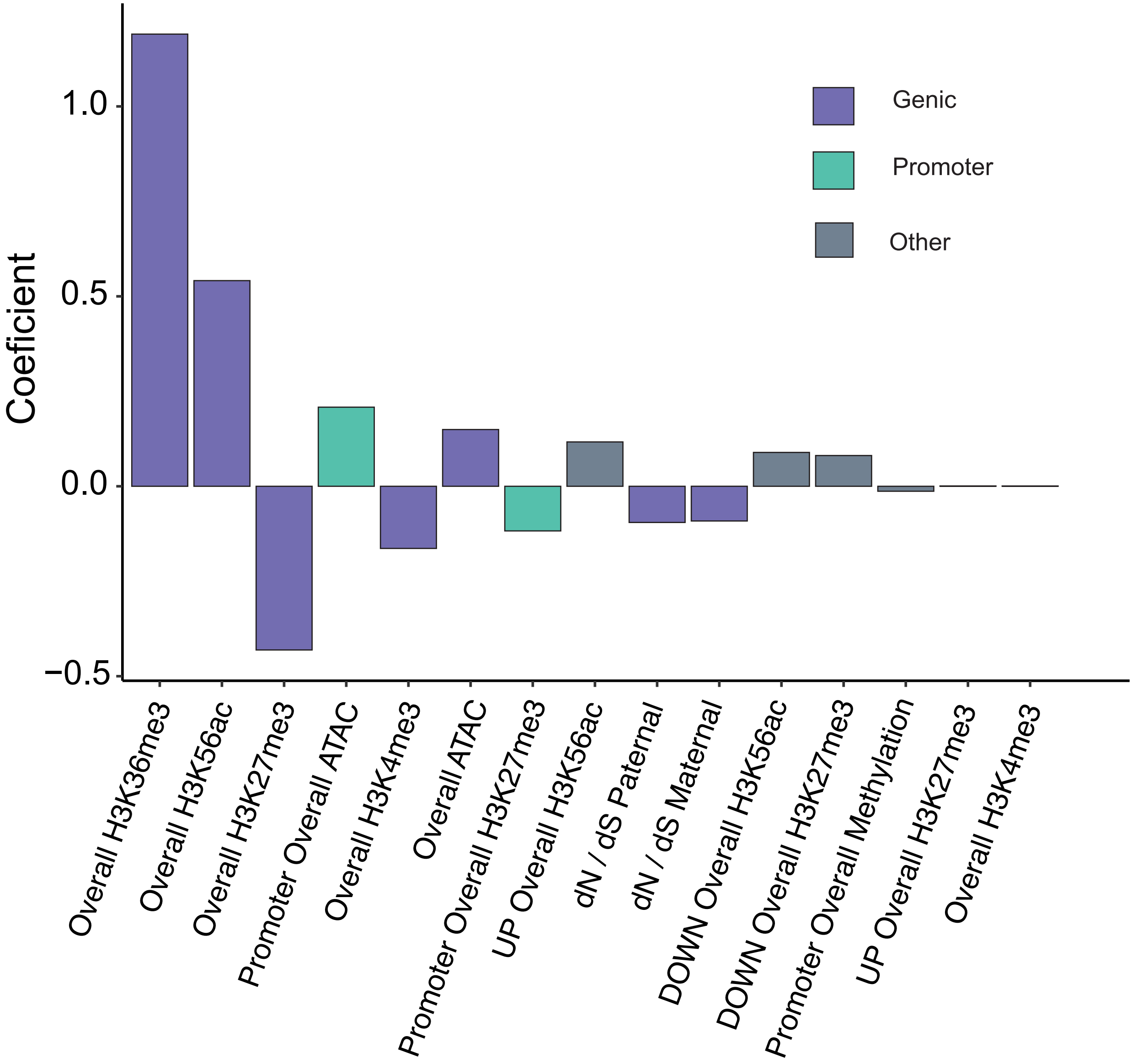

Supplemental Fig. S7. Modeling of overall gene expression of all genes in the 'Fairchild' genome after increasing shrinkage parameter (λ). Coefficients for significant predictors of overall expression (log2(TPM)) of all genes (n = 30,724) ordered by magnitude (R = 64.71). Factors are partitioned by genomic region and colored to indicate whether they reside in genes, promoters (1kb of TSS), or upstream/ downstream putative regulatory regions of the focal gene (ACRs present 5 Kb upstream of promoter / 5 Kb downstream of gene). A summary of all models used is available in additional file 1: Table S12.

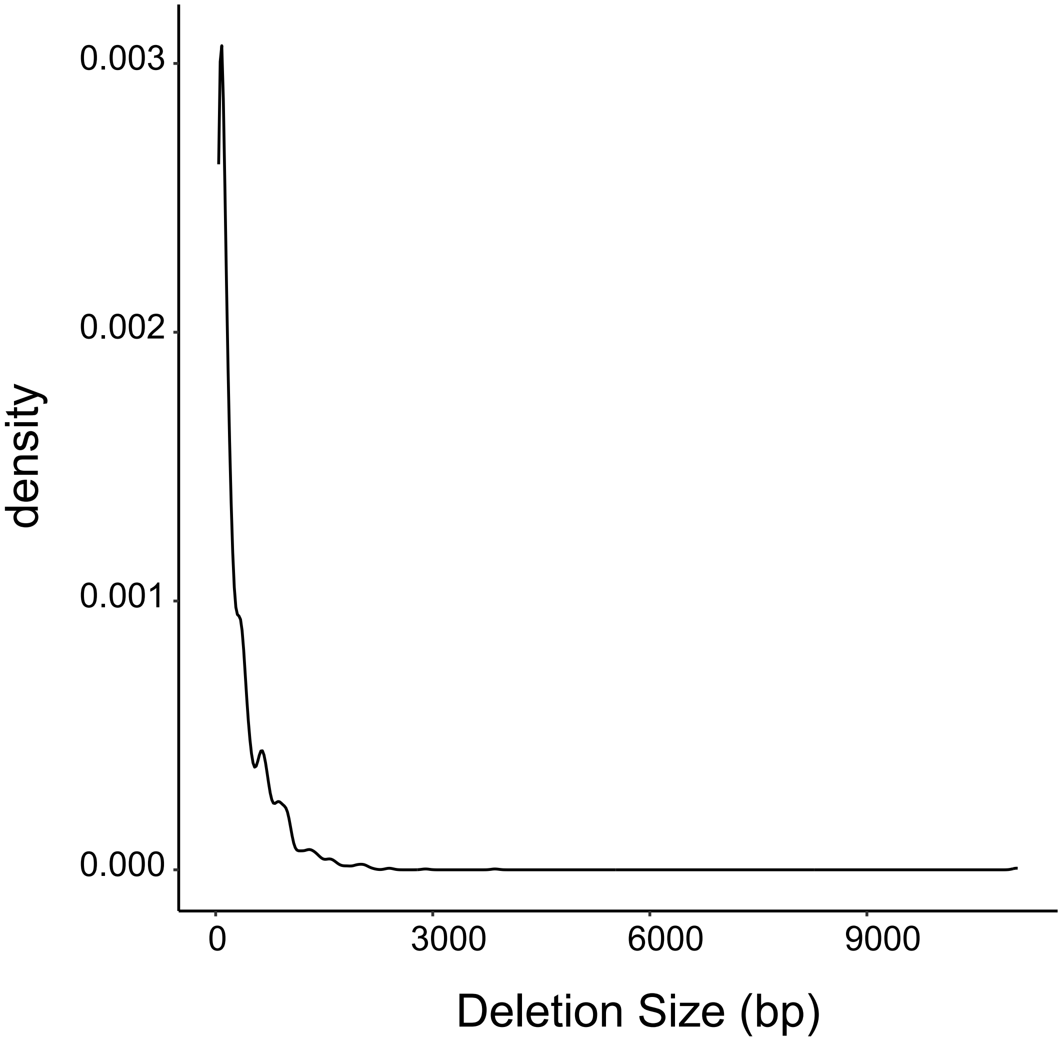

Supplemental Fig. S8. Distribution of size of deletions detected using 10x Genomics linked-reads (n = 2,463).

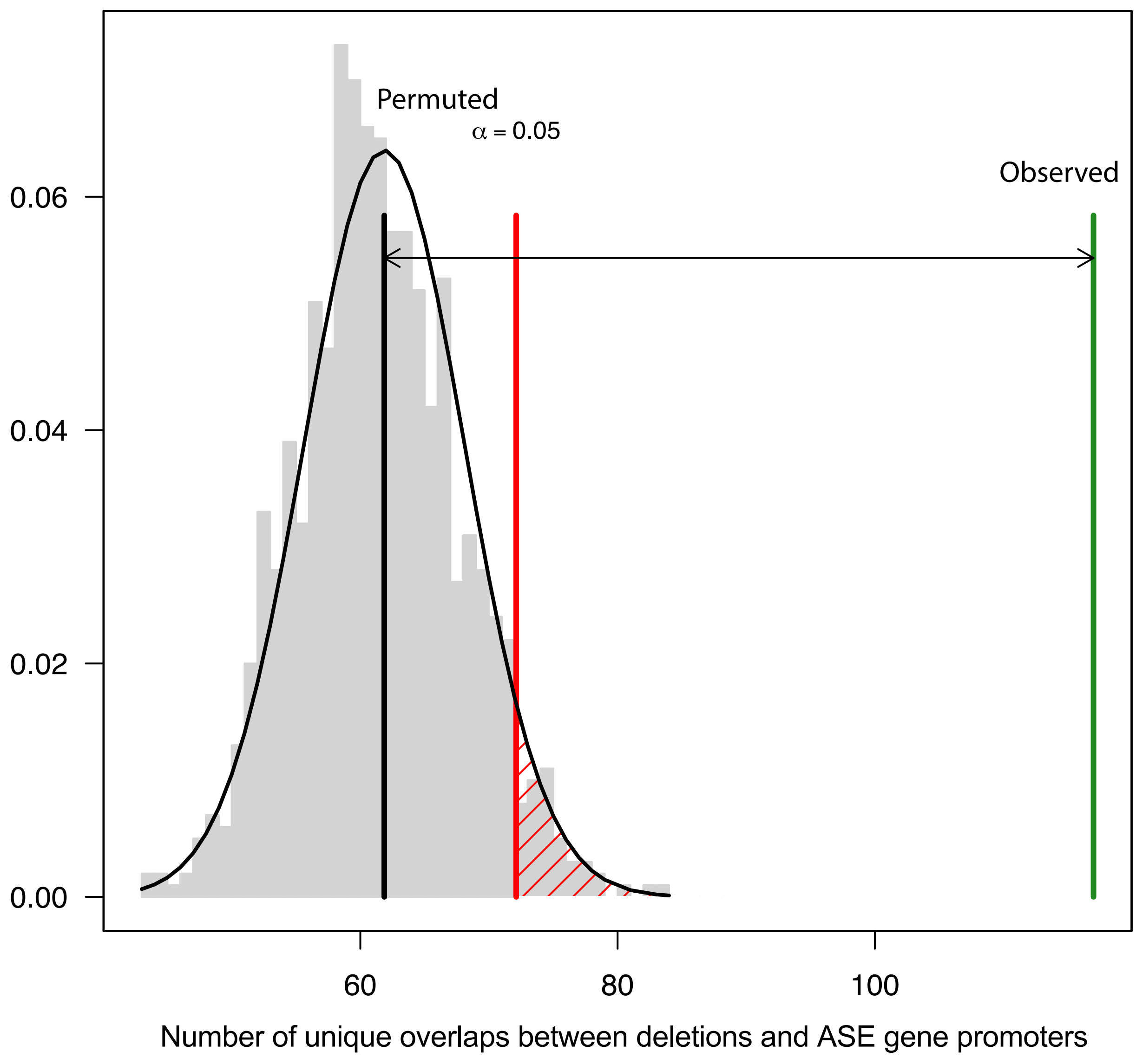

Supplemental Fig. S9. Promoters of ASE genes are enriched for deletions. The number of unique overlaps between promoters of ASE genes and deletions (green vertical line) was compared to 1,000 permutations of promoters from all genes in the Fairchild genome ($p < 0.001$). The black vertical line indicates the mean of the 1,000 permutations and the red vertical line indicates a significance threshold ($p = 0.05$).

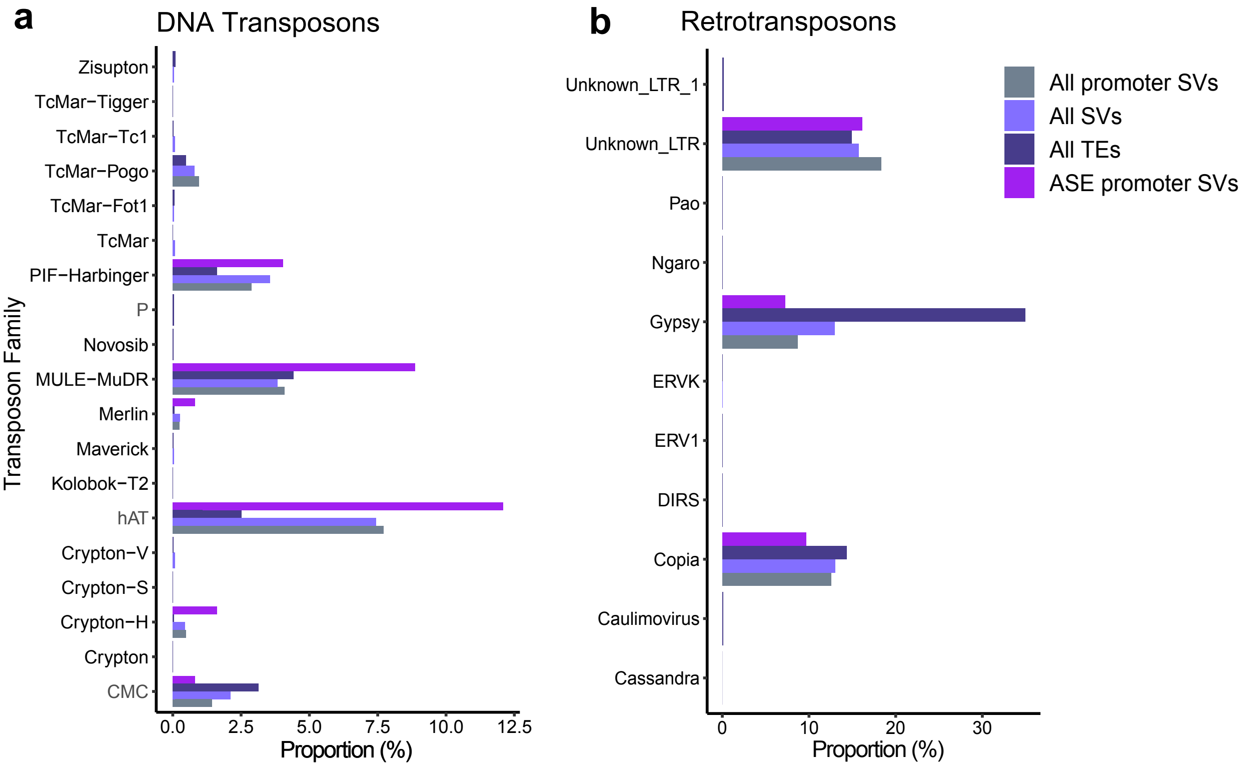

Supplemental Fig. S10. Transposable element content of structural variation in gene promoters. a) The proportion of either: all promoters, all structural variants (deletions), all annotated transposable elements, or structural variants in promoters of ASE genes that belong to specific DNA transposon families or retrotransposons (b).

a

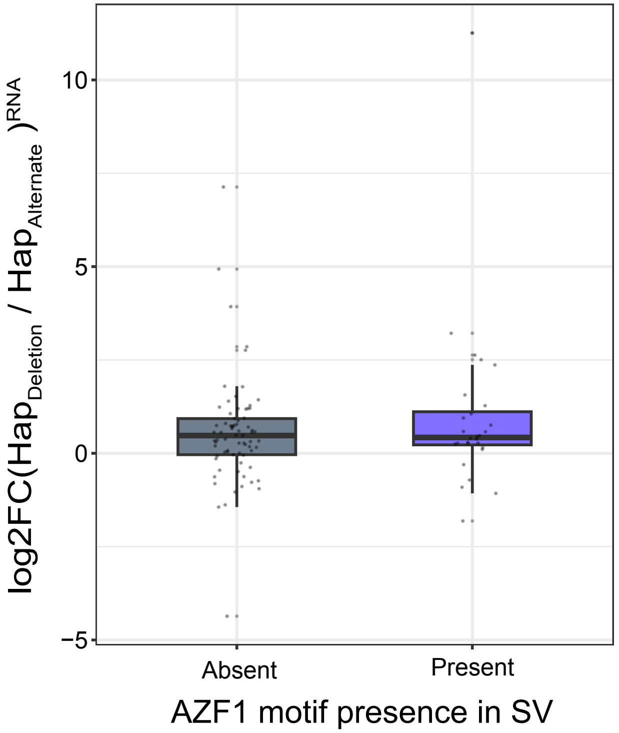

b

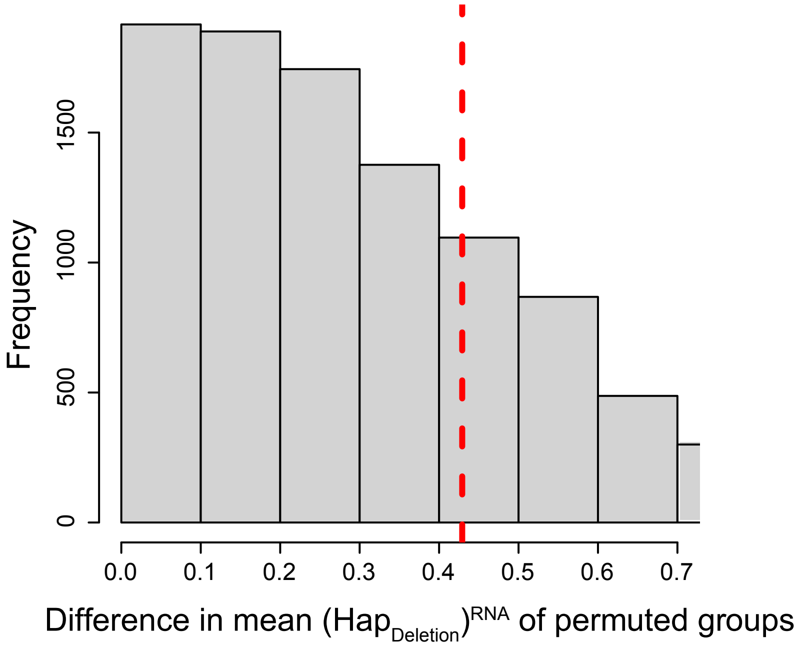

Supplemental Fig. S11. AZF1 motif occurrence in structural variants within promoters of ASE genes. a) A motif enrichment analysis of sequence removed in the ‘deleted’ allele of 104 ASE genes with deletions in their promoter identified four significantly enriched motifs, with the most abundant (n=28) matching the binding site of zinc-finger protein 1 (AZF1), a zinc-finger protein that acts as a transcriptional repressor (Kodaira et al. 2011). Comparison of allele-specific expression of the deletion containing allele ((HapDeletion)RNA) of genes with the motif in the ‘deleted’ allele (n=28) compared to those without the motif (n = 78) indicates that there is not a significant effect of motif presence on allele expression (10,000 permutations, p = 0.27). b) Distribution of the difference in mean expression of the deletion containing allele ((HapDeletion)RNA) for 10,000 permuted groups of genes selected from the 104 ASE genes with promoter deletions. The red dotted-line represents the observed difference in means between genes with the AZF1 motif in the deleted allele versus those without the motif.

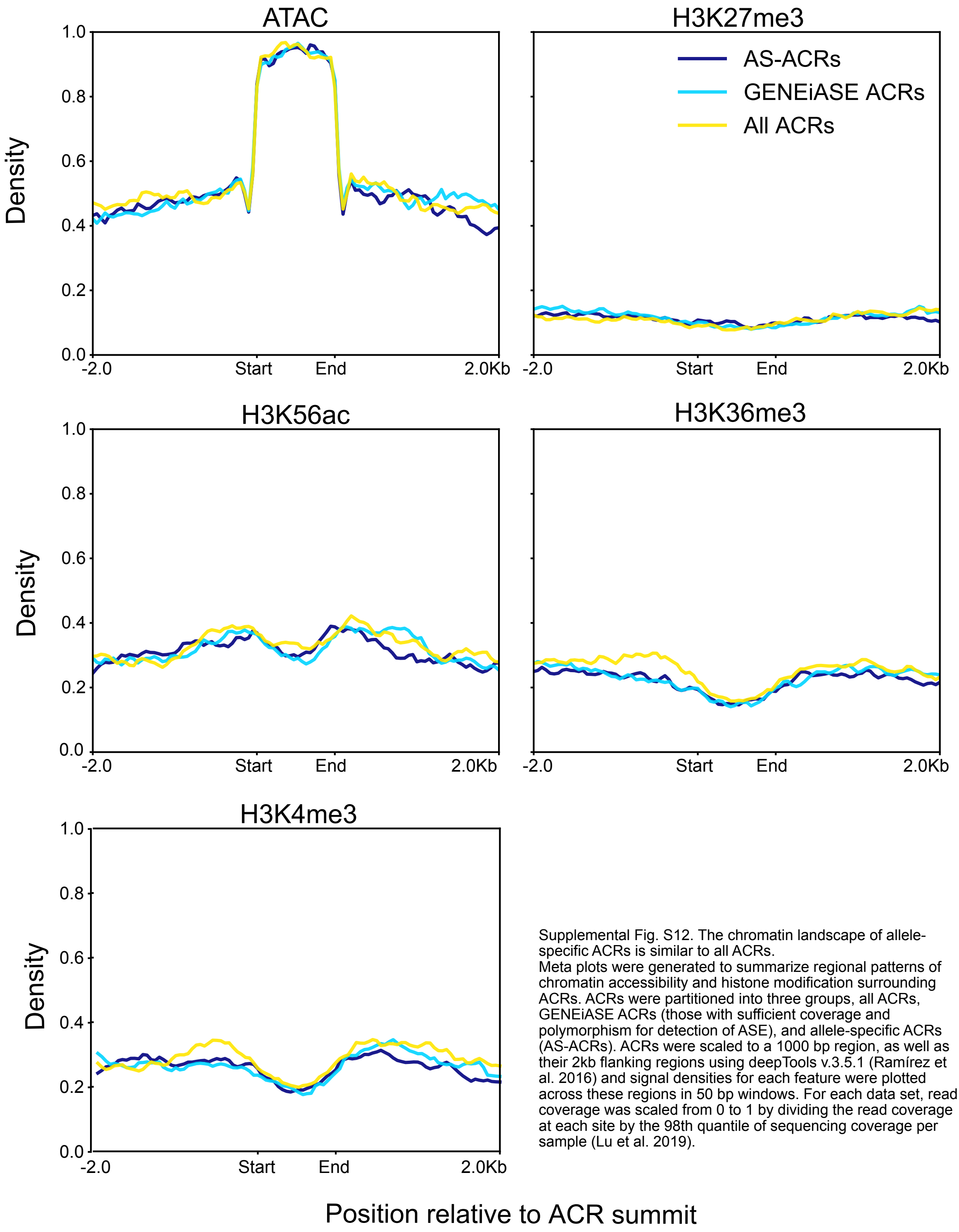

Supplemental Fig. S12. The chromatin landscape of allele-specific ACRs is similar to all ACRs.
Meta plots were generated to summarize regional patterns of chromatin accessibility and histone modification surrounding ACRs. ACRs were partitioned into three groups, all ACRs, GENEiASE ACRs (those with sufficient coverage and polymorphism for detection of ASE), and allele-specific ACRs (AS-ACRs). ACRs were scaled to a 1000 bp region, as well as their 2kb flanking regions using deepTools v.3.5.1 (Ramírez et al. 2016) and signal densities for each feature were plotted across these regions in 50 bp windows. For each data set, read coverage was scaled from 0 to 1 by dividing the read coverage at each site by the 98th quantile of sequencing coverage per sample (Lu et al. 2019).

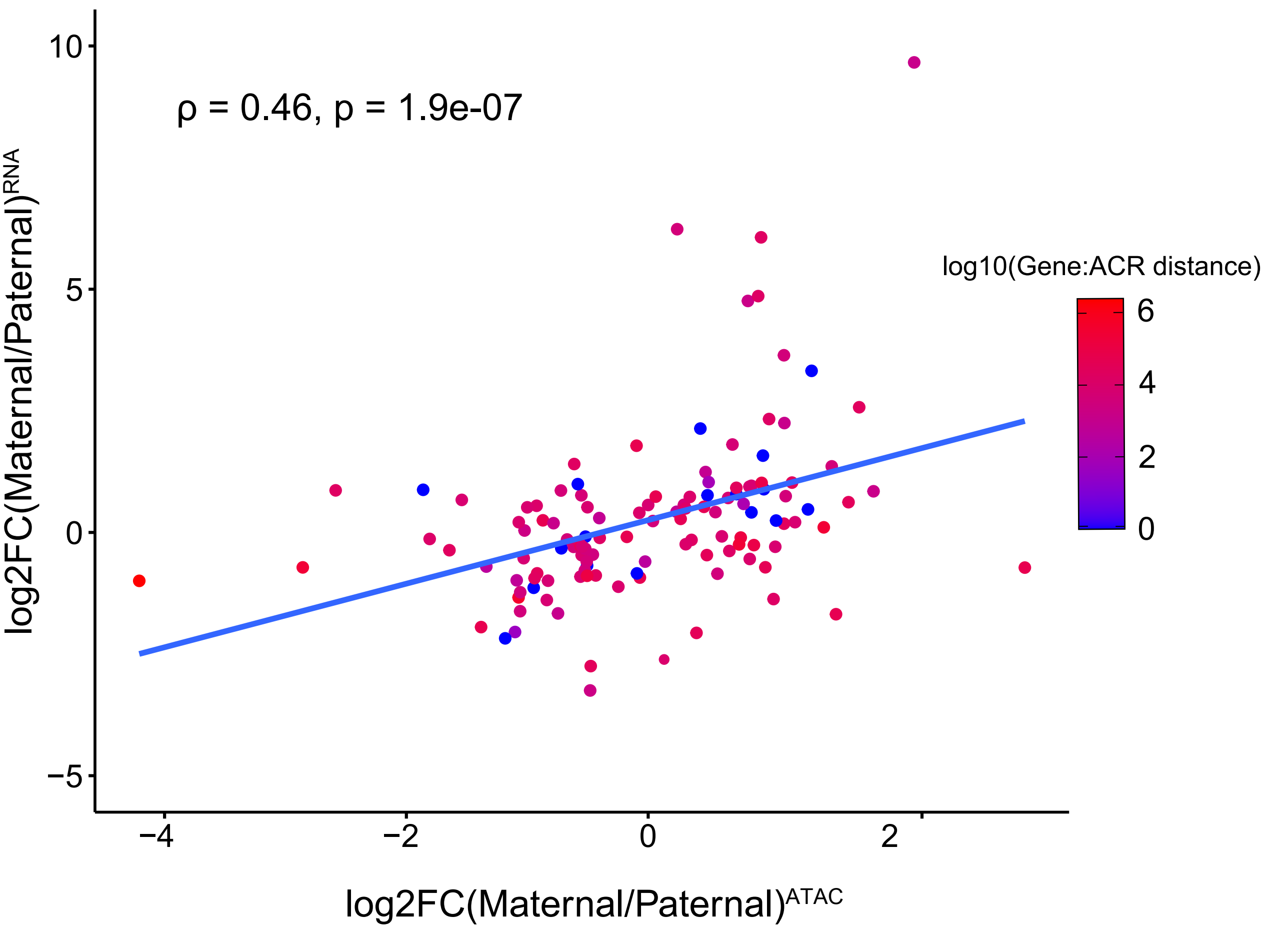

Supplemental Fig. S13. The relationship between allele-accessibility of AS-ACRs and allele-expression of ASE genes. AS-ACRs were paired with the nearest ASE gene if they were present in the same phase-block (n=122, median distance = 7447.5 bp). Allele-accessibility of AS-ACRs represented as the ratio of maternal : paternal ATAC-seq reads is positively correlated with allele-specific expression of neighboring genes (R = 0.46 , p = 1.9e-07). Points are colored by the distance between the AS-ACRs and its nearest ASE gene.

a

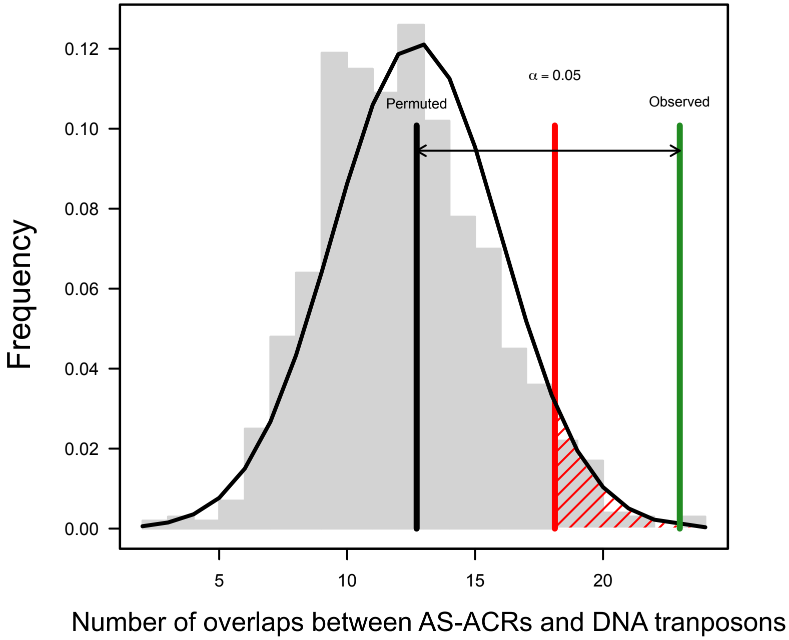

b

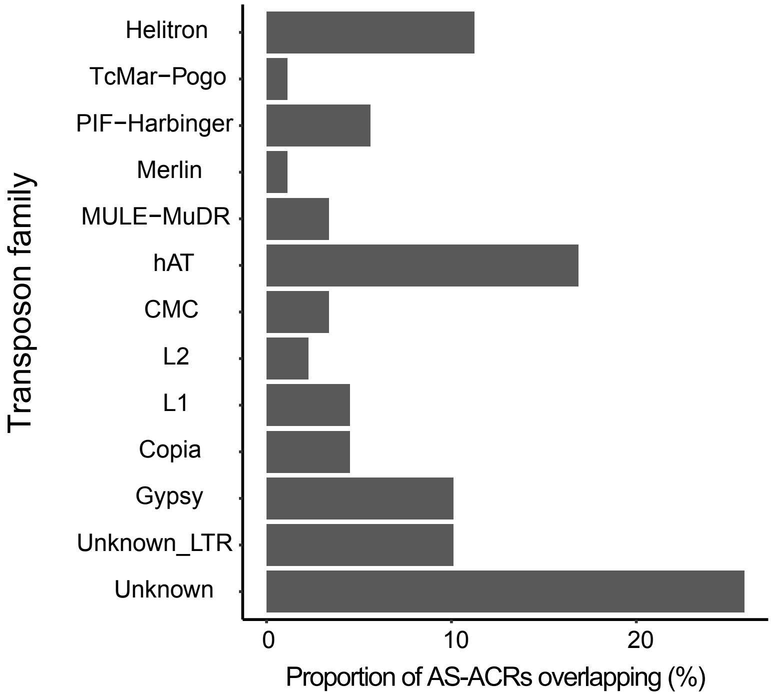

Supplemental Fig. S14. AS-ACRs are enriched for DNA transposons. a) The number of unique overlaps between AS-ACRs and DNA transposons (green vertical line) was compared to 1,000 permutations from all ACRs in the Fairchild genome (p = 0.007). The black vertical line indicates the mean of the 1,000 permutations and the red vertical line indicates a significance threshold (p = 0.05). b) The proportion of AS-ACRs overlapping transposons belonging to specific families.

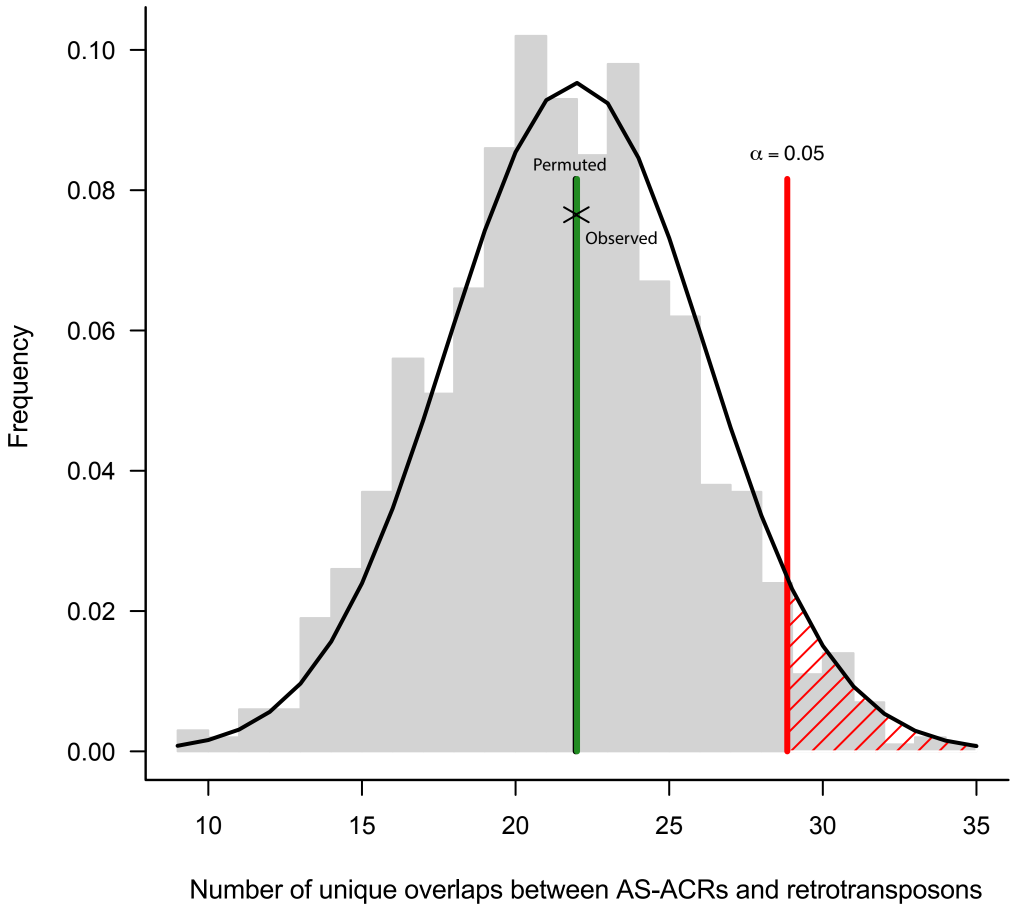

Supplemental Fig. S15. AS-ACRs are not enriched for retrotransposons. a) The number of unique overlaps between AS-ACRs and retrotransposons (green vertical line) was compared to 1,000 permutations from all ACRs in the Fairchild genome ($p = 0.5345$). The black vertical line indicates the mean of the 1,000 permutations and the red vertical line indicates a significance threshold ($p = 0.05$).

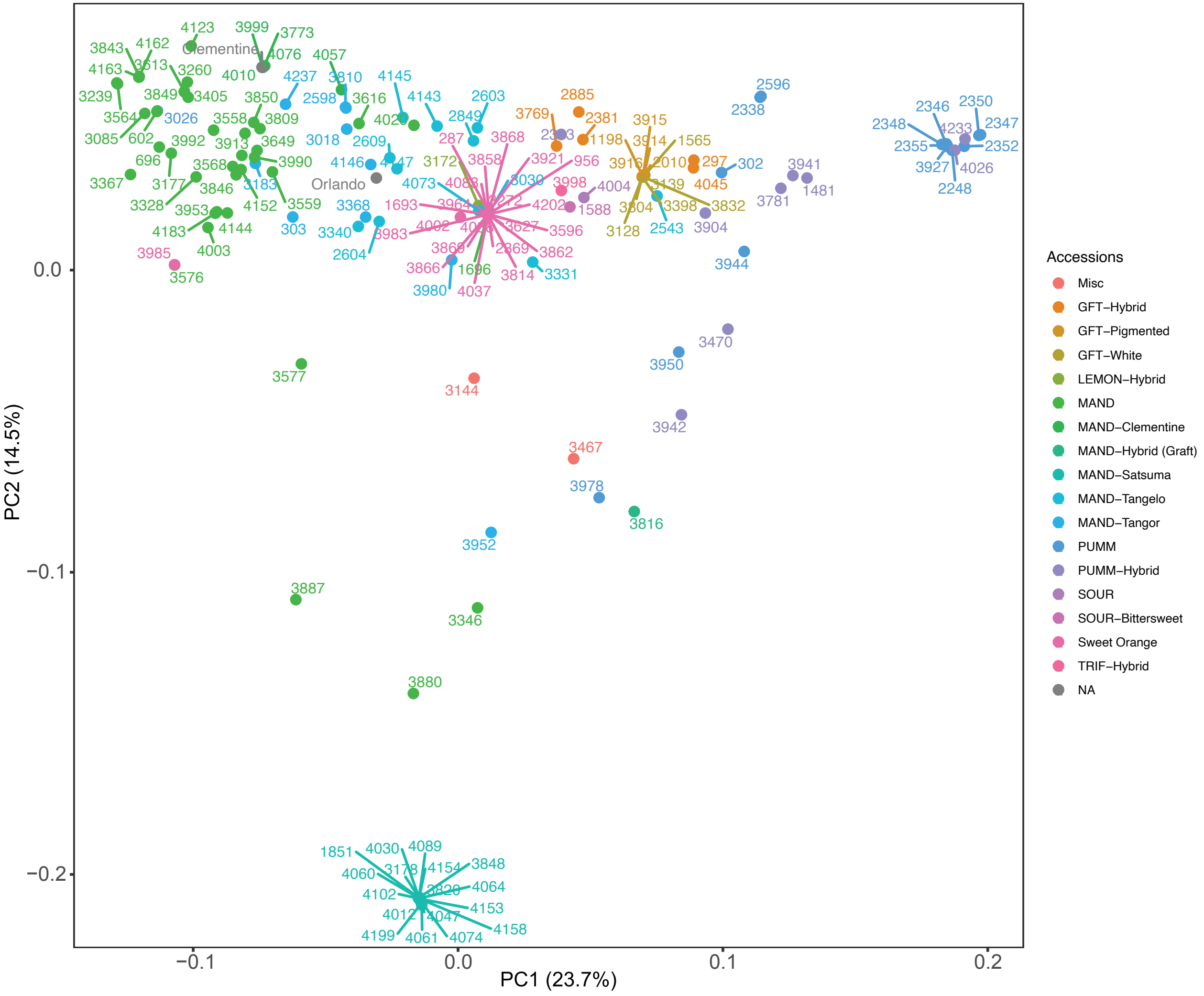

Supplemental Fig. S16. Population structure of the diversity panel of 154 citrus accessions used for GWAS. The first two principal components representing genetic diversity were calculated using genome-wide SNPs. Eigenvectors and eigenvalues were calculated from a subset of 94,885 SNPs from the 2.4 million SNPs dataset after pruning with plink v1.90b6.24 using parameters "--indep-pairwise 50 10 0.2".

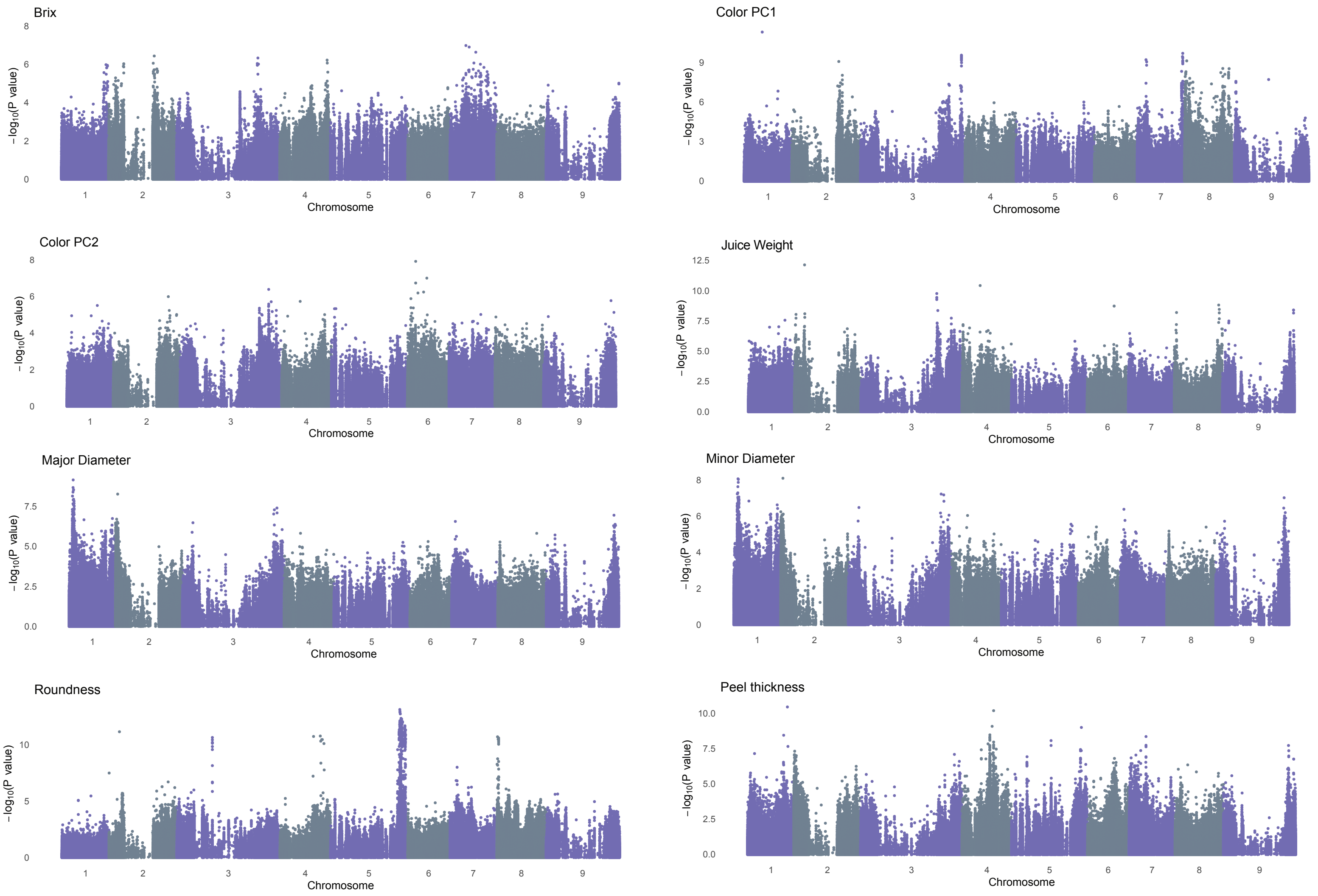

Fig. S17: Manhattan plots for the fifteen traits tested using GWAS, the dotted line represents a Bonferroni-corrected significance threshold (α = 0.05) for the 2.24 million SNPs tested. The phenotype is shown in the top left corner of each plot.

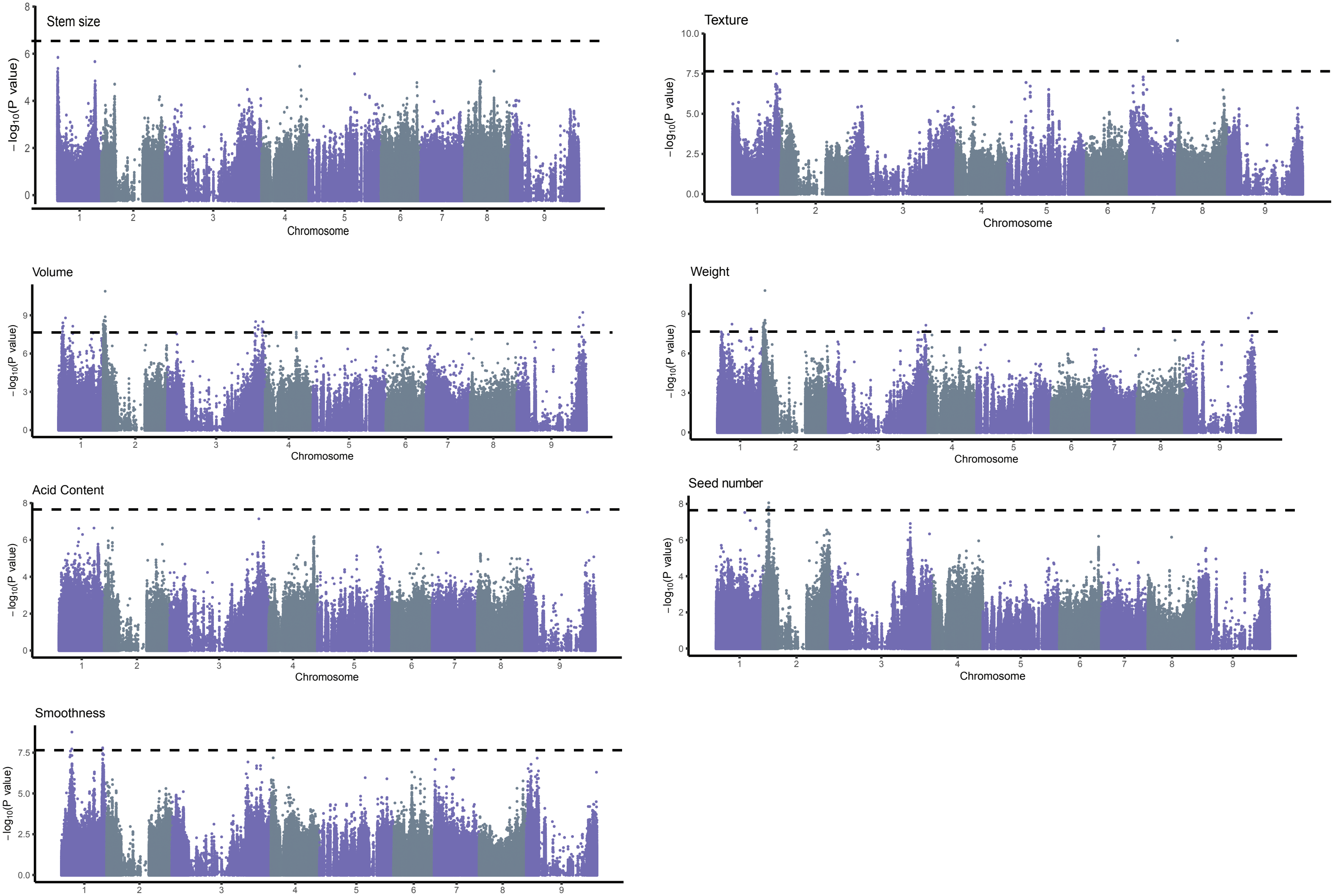

Fig. S17 : Manhattan plots for the fifteen traits tested using GWAS, the dotted line represents a Bonferroni-corrected significance threshold (α = 0.05) for the 2.24 million SNPs tested. The phenotype is shown in the top left corner of each plot.

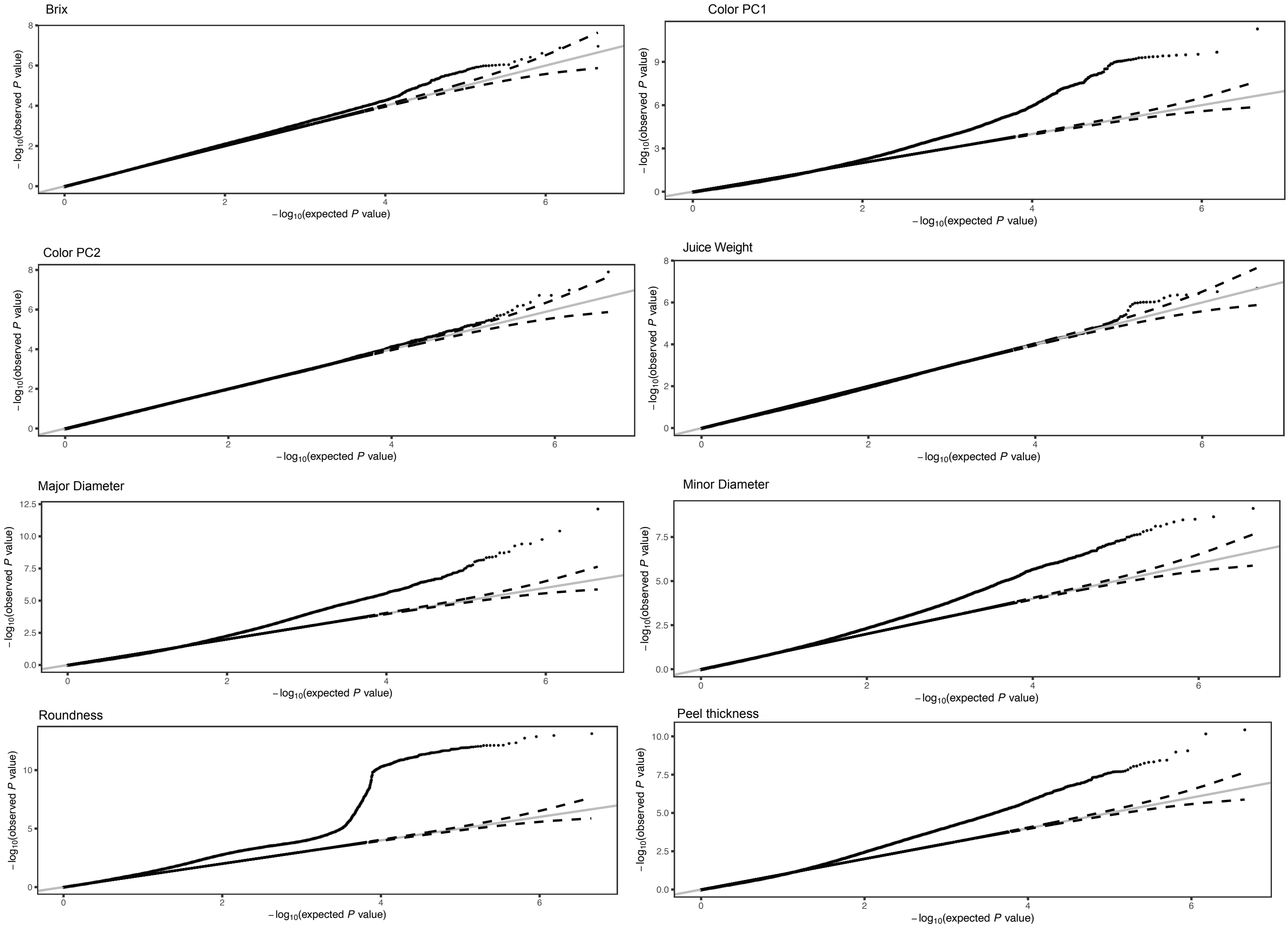

Fig. S18: Quantile-quantile plots indicating observed and expected p-values for a linear-mixed model of each trait. Dotted lines represent the 95% confidence interval.

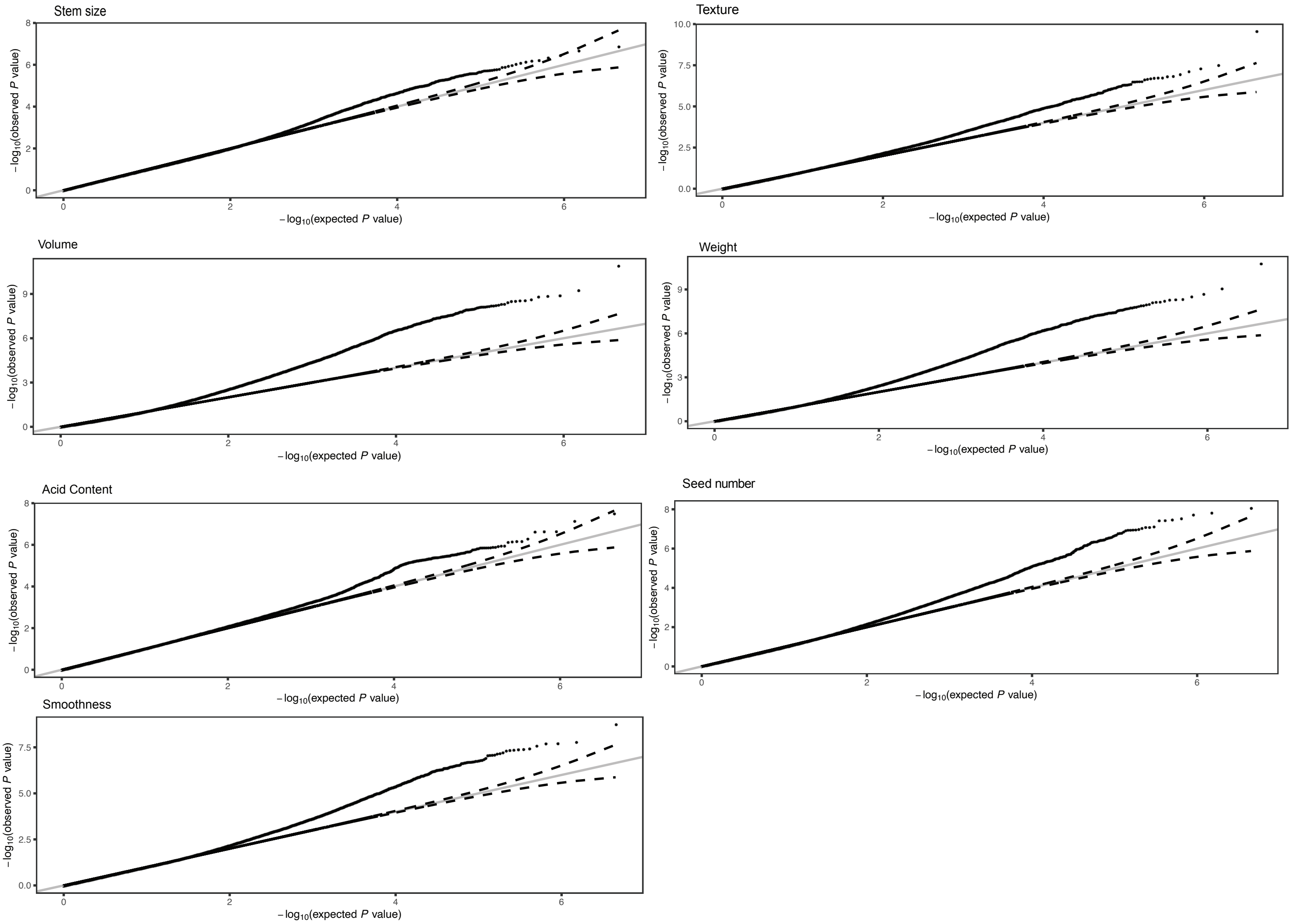

Fig. S18: Quantile-quantile plots indicating observed and expected p-values for a linear-mixed model of each trait. Dotted lines represent the 95% confidence interval.

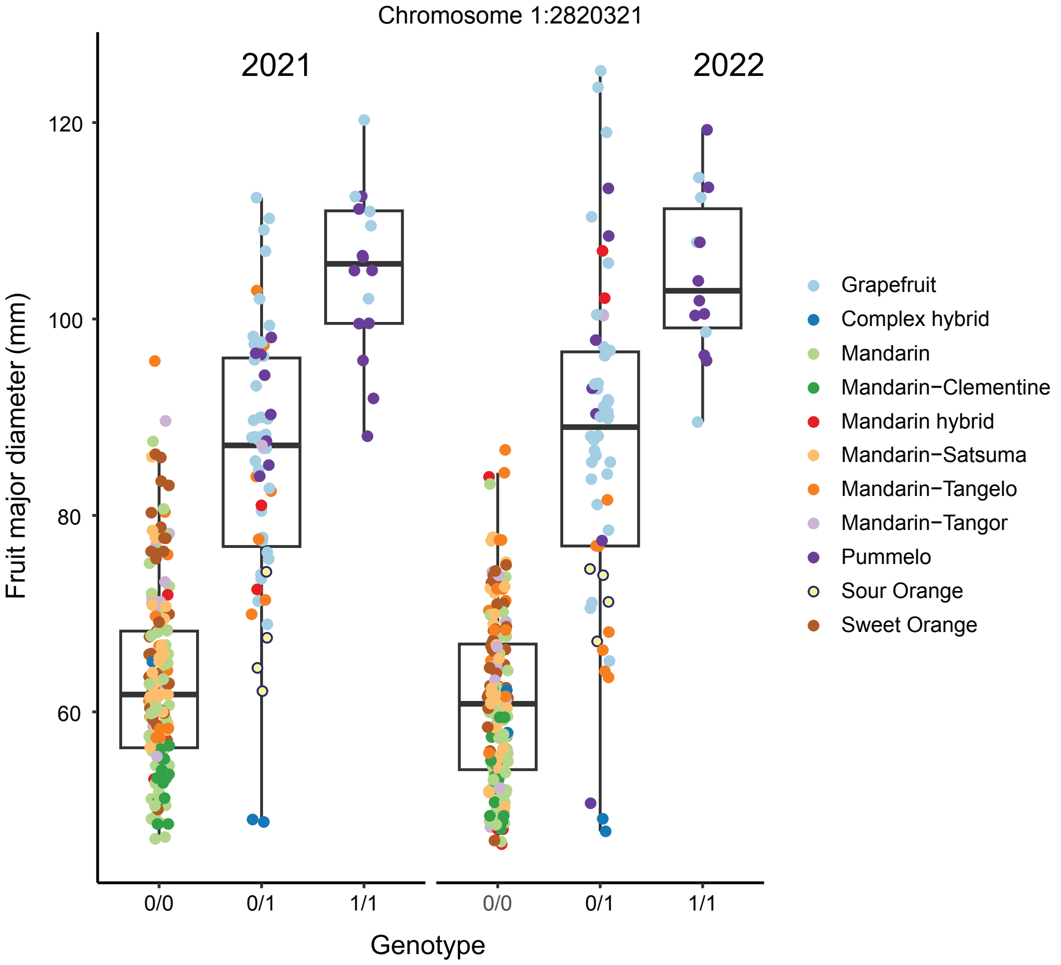

Fig. S19: Mean major diameter of fruits of accessions that segregate at the lead SNP (chr1:2820321). Points are colored by the "market type" of each accession. "Complex hybrid" includes individuals with ancestry from mandarin (C.reticulata), pummelo (C.maxima), and additional citrus species such as trifoliate orange (Poncirus trifoliata). Mandarins with known subclassifications such as satsumas, tangelos, clementines, and tangors are labeled. Each point represents the mean of all fruits from an individual tree, with observations for both 2021 and 2022 harvests.

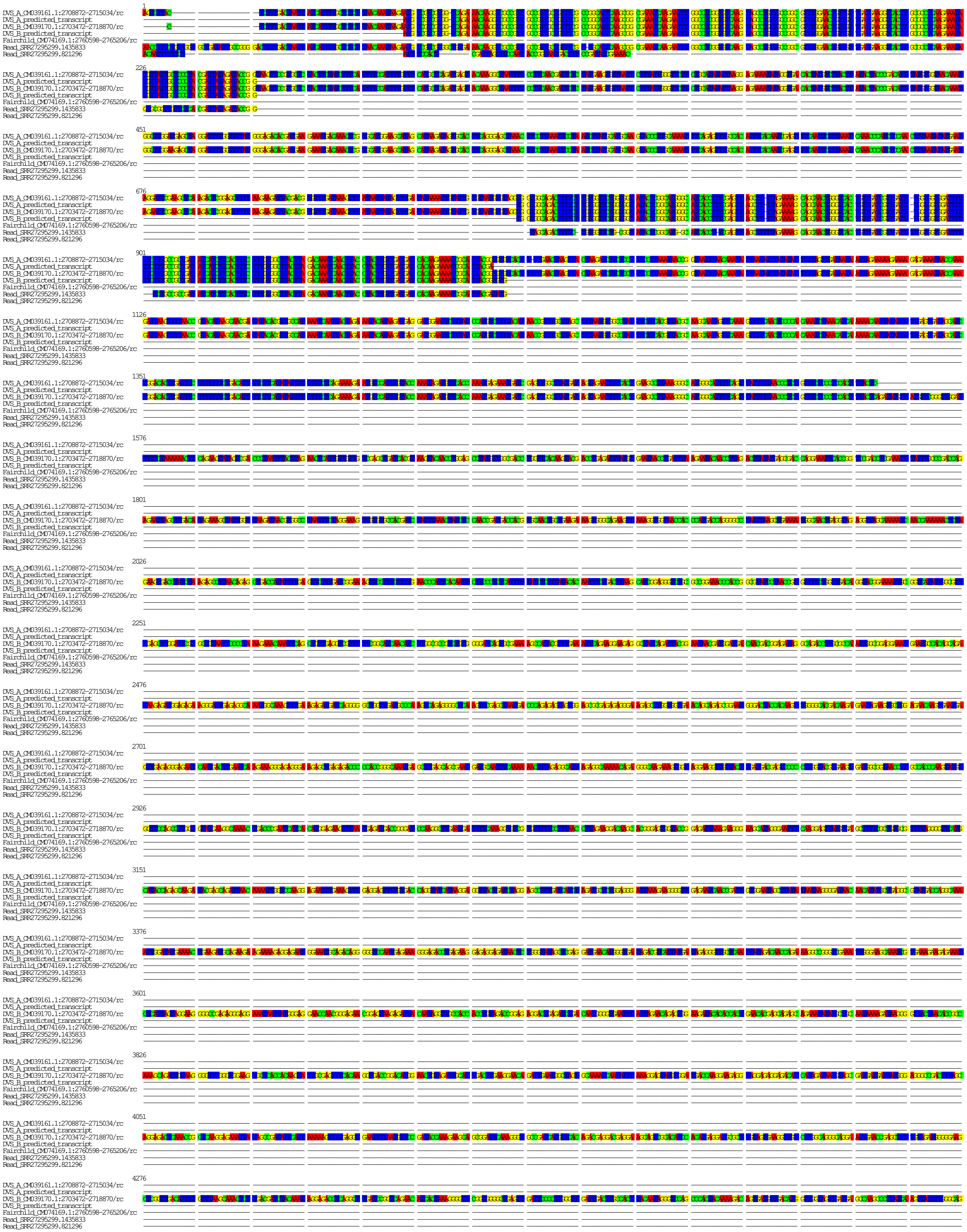
DVS_A_CM039161.1:2708872-2715034/rc
DVS_A_predicted_transcript
DVS_B_CM039170.1:2703472-2718870/rc
DVS_B_predicted_transcript
Fairchild_CM074169.1:2760598-2765206/rc
Read_SRR27295299.1435833
Read_SRR27295299.821296
1
226
451
676
901
1126
1351
1576
1801
2026
2251
2476
2701
2926
3151
3376
3601
3826
4051
4276

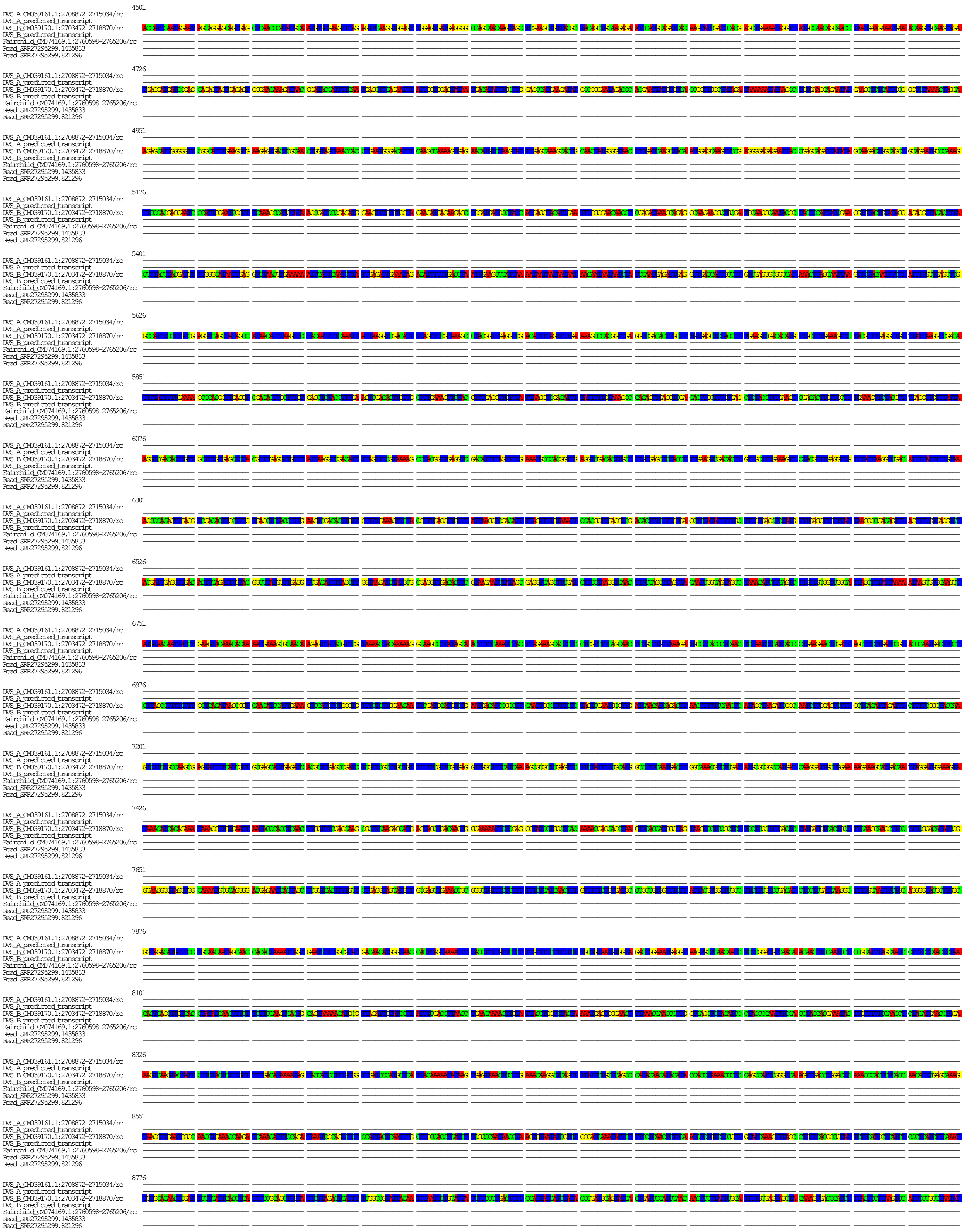

4501
DVS_A_CM039161.1:2708872-2715034/rc
DVS_A_predicted_transcript
DVS_B_CM039170.1:2703472-2718870/rc
DVS_B_predicted_transcript
Fairchild_CM074169.1:2760598-2765206/rc
Read_SRR27295299.1435833
Read_SRR27295299.821296
4726
DVS_A_CM039161.1:2708872-2715034/rc
DVS_A_predicted_transcript
DVS_B_CM039170.1:2703472-2718870/rc
DVS_B_predicted_transcript
Fairchild_CM074169.1:2760598-2765206/rc
Read_SRR27295299.1435833
Read_SRR27295299.821296
4951
DVS_A_CM039161.1:2708872-2715034/rc
DVS_A_predicted_transcript
DVS_B_CM039170.1:2703472-2718870/rc
DVS_B_predicted_transcript
Fairchild_CM074169.1:2760598-2765206/rc
Read_SRR27295299.1435833
Read_SRR27295299.821296
5176
DVS_A_CM039161.1:2708872-2715034/rc
DVS_A_predicted_transcript
DVS_B_CM039170.1:2703472-2718870/rc
DVS_B_predicted_transcript
Fairchild_CM074169.1:2760598-2765206/rc
Read_SRR27295299.1435833
Read_SRR27295299.821296
5401
DVS_A_CM039161.1:2708872-2715034/rc
DVS_A_predicted_transcript
DVS_B_CM039170.1:2703472-2718870/rc
DVS_B_predicted_transcript
Fairchild_CM074169.1:2760598-2765206/rc
Read_SRR27295299.1435833
Read_SRR27295299.821296
5626
DVS_A_CM039161.1:2708872-2715034/rc
DVS_A_predicted_transcript
DVS_B_CM039170.1:2703472-2718870/rc
DVS_B_predicted_transcript
Fairchild_CM074169.1:2760598-2765206/rc
Read_SRR27295299.1435833
Read_SRR27295299.821296
5851
DVS_A_CM039161.1:2708872-2715034/rc
DVS_A_predicted_transcript
DVS_B_CM039170.1:2703472-2718870/rc
DVS_B_predicted_transcript
Fairchild_CM074169.1:2760598-2765206/rc
Read_SRR27295299.1435833
Read_SRR27295299.821296
6076
DVS_A_CM039161.1:2708872-2715034/rc
DVS_A_predicted_transcript
DVS_B_CM039170.1:2703472-2718870/rc
DVS_B_predicted_transcript
Fairchild_CM074169.1:2760598-2765206/rc
Read_SRR27295299.1435833
Read_SRR27295299.821296
6301
DVS_A_CM039161.1:2708872-2715034/rc
DVS_A_predicted_transcript
DVS_B_CM039170.1:2703472-2718870/rc
DVS_B_predicted_transcript
Fairchild_CM074169.1:2760598-2765206/rc
Read_SRR27295299.1435833
Read_SRR27295299.821296
6526
DVS_A_CM039161.1:2708872-2715034/rc
DVS_A_predicted_transcript
DVS_B_CM039170.1:2703472-2718870/rc
DVS_B_predicted_transcript
Fairchild_CM074169.1:2760598-2765206/rc
Read_SRR27295299.1435833
Read_SRR27295299.821296
6751
DVS_A_CM039161.1:2708872-2715034/rc
DVS_A_predicted_transcript
DVS_B_CM039170.1:2703472-2718870/rc
DVS_B_predicted_transcript
Fairchild_CM074169.1:2760598-2765206/rc
Read_SRR27295299.1435833
Read_SRR27295299.821296
6976
DVS_A_CM039161.1:2708872-2715034/rc
DVS_A_predicted_transcript
DVS_B_CM039170.1:2703472-2718870/rc
DVS_B_predicted_transcript
Fairchild_CM074169.1:2760598-2765206/rc
Read_SRR27295299.1435833
Read_SRR27295299.821296
7201
DVS_A_CM039161.1:2708872-2715034/rc
DVS_A_predicted_transcript
DVS_B_CM039170.1:2703472-2718870/rc
DVS_B_predicted_transcript
Fairchild_CM074169.1:2760598-2765206/rc
Read_SRR27295299.1435833
Read_SRR27295299.821296
7426
DVS_A_CM039161.1:2708872-2715034/rc
DVS_A_predicted_transcript
DVS_B_CM039170.1:2703472-2718870/rc
DVS_B_predicted_transcript
Fairchild_CM074169.1:2760598-2765206/rc
Read_SRR27295299.1435833
Read_SRR27295299.821296
7651
DVS_A_CM039161.1:2708872-2715034/rc
DVS_A_predicted_transcript
DVS_B_CM039170.1:2703472-2718870/rc
DVS_B_predicted_transcript
Fairchild_CM074169.1:2760598-2765206/rc
Read_SRR27295299.1435833
Read_SRR27295299.821296
7876
DVS_A_CM039161.1:2708872-2715034/rc
DVS_A_predicted_transcript
DVS_B_CM039170.1:2703472-2718870/rc
DVS_B_predicted_transcript
Fairchild_CM074169.1:2760598-2765206/rc
Read_SRR27295299.1435833
Read_SRR27295299.821296
8101
DVS_A_CM039161.1:2708872-2715034/rc
DVS_A_predicted_transcript
DVS_B_CM039170.1:2703472-2718870/rc
DVS_B_predicted_transcript
Fairchild_CM074169.1:2760598-2765206/rc
Read_SRR27295299.1435833
Read_SRR27295299.821296
8326
DVS_A_CM039161.1:2708872-2715034/rc
DVS_A_predicted_transcript
DVS_B_CM039170.1:2703472-2718870/rc
DVS_B_predicted_transcript
Fairchild_CM074169.1:2760598-2765206/rc
Read_SRR27295299.1435833
Read_SRR27295299.821296
8551
DVS_A_CM039161.1:2708872-2715034/rc
DVS_A_predicted_transcript
DVS_B_CM039170.1:2703472-2718870/rc
DVS_B_predicted_transcript
Fairchild_CM074169.1:2760598-2765206/rc
Read_SRR27295299.1435833
Read_SRR27295299.821296
8776
DVS_A_CM039161.1:2708872-2715034/rc
DVS_A_predicted_transcript
DVS_B_CM039170.1:2703472-2718870/rc
DVS_B_predicted_transcript
Fairchild_CM074169.1:2760598-2765206/rc
Read_SRR27295299.1435833
Read_SRR27295299.821296

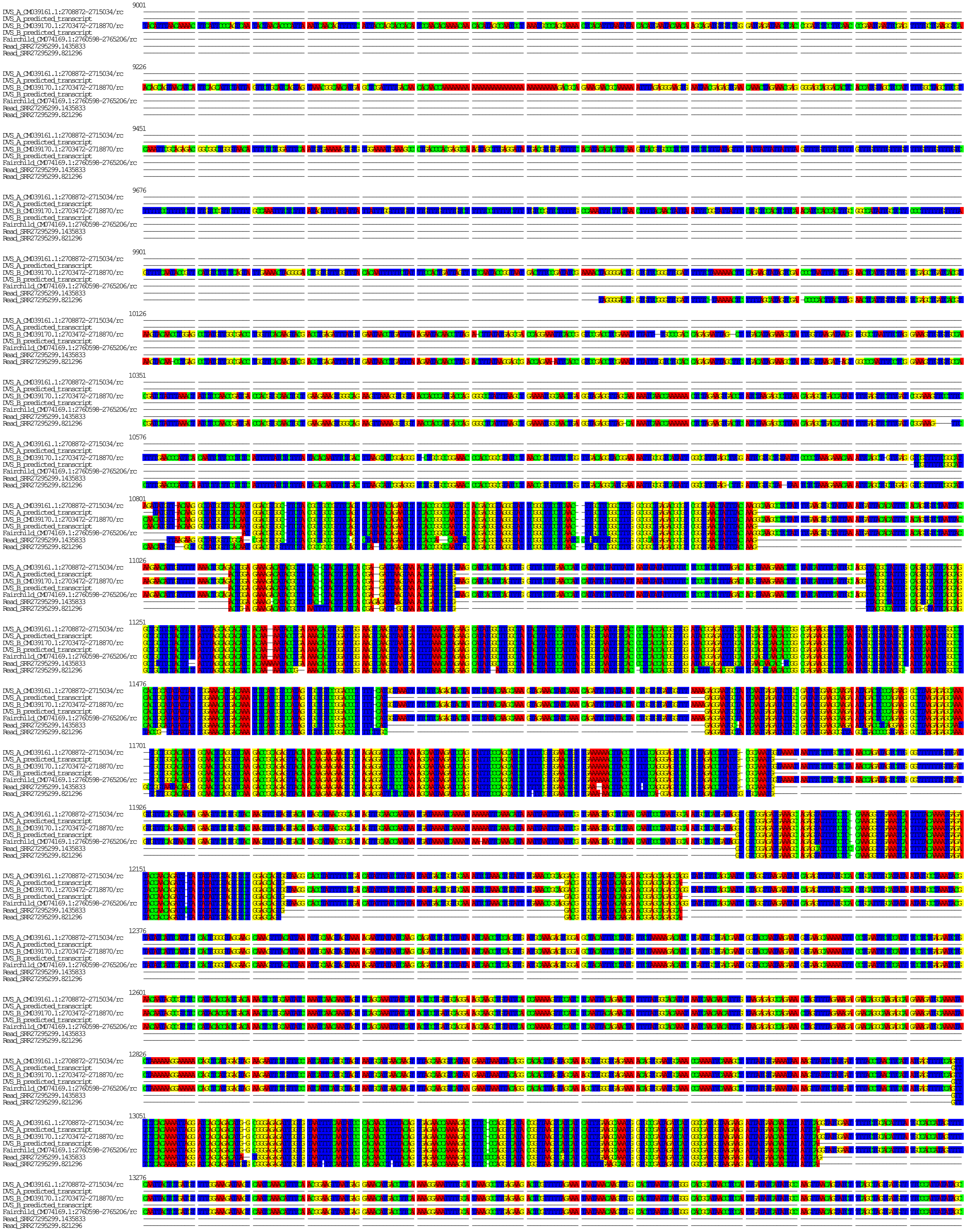
9001
9226
9451
9676
9901
10126
10351
10576
10801
11026
11251
11476
11701
11926
12151
12376
12601
12826
13051
13276
DVS_A_CM039161.1:2708872-2715034/rc
DVS_A_predicted_transcript
DVS_B_CM039170.1:2703472-2718870/rc
DVS_B_predicted_transcript
Fairchild_CM074169.1:2760598-2765206/rc
Read_SRR27295299.1435833
Read_SRR27295299.821296

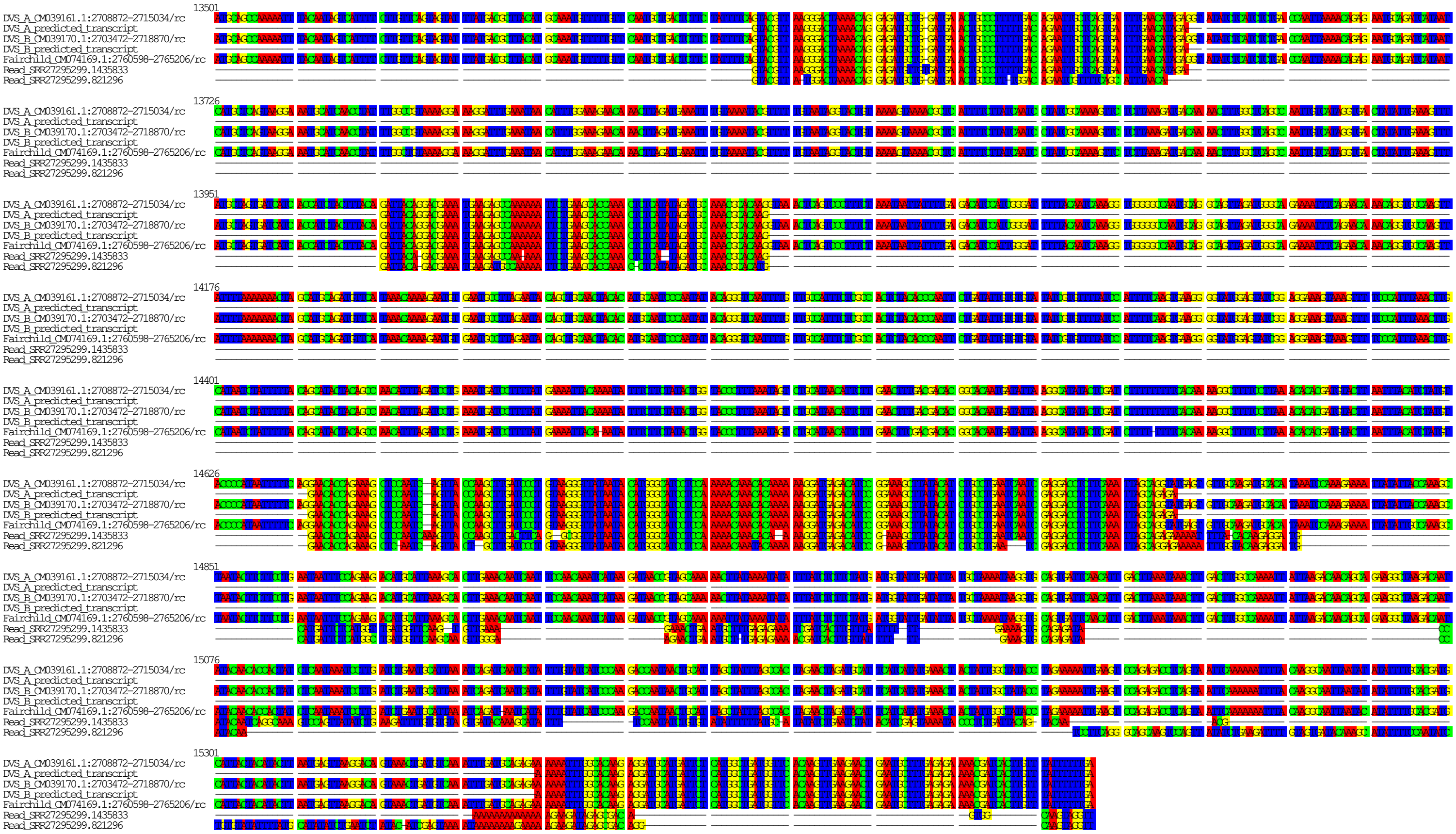

Fig. S20: Nucleotide alignment of CitKC1. The alignment includes the following sequences: 1) the genomic sequence for CitKC1 from ‘Valencia’ sweet orange and ‘Fairchild’ mandarin, 2) the predicted transcripts for CitKC1 from sweet orange (both the SV and + allele), and raw FL-cDNA ONT reads from sweet orange (SRR27295299). The alignment was generated by MUSCLE and visualized in Seaview. DVS A and DVS B correspond to haplotypes A and B from ‘Valencia’, respectively.

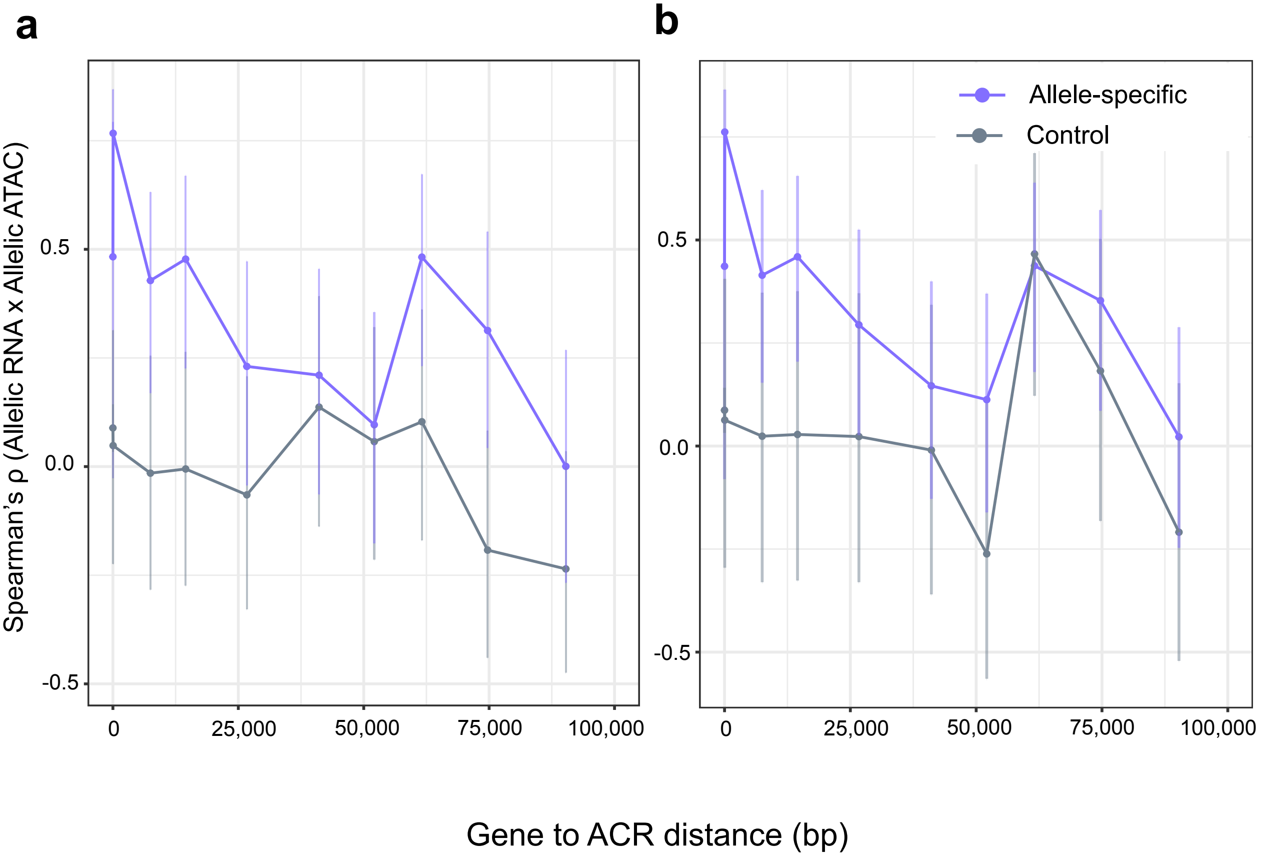

Fig. S21: The relationship between allele-accessibility of AS-ACRs and allele-expression of ASE genes decays over longer distances. a) ASE genes were paired with their nearest AS-ACR and only ACR-gene pairs within the same phase block were retained. ACR-gene pairs were then split into 25 quantiles based on the distance between gene and ACR. For each quantile, the spearman correlation coefficient (rho) of allele-accessibility and allele-expression of gene-ACR pairs (n=30) was calculated. As a control, ASE genes were paired with their nearest ACR, regardless of whether the ACR has significant allele-specificity. The spearman correlation coefficient was calculated for control ACR-gene pairs (n=30), and we reported the average of 1000 permutations of ACR-gene pairs. The vertical lines indicate the 95% confidence interval for the spearman correlation coefficient (rho).

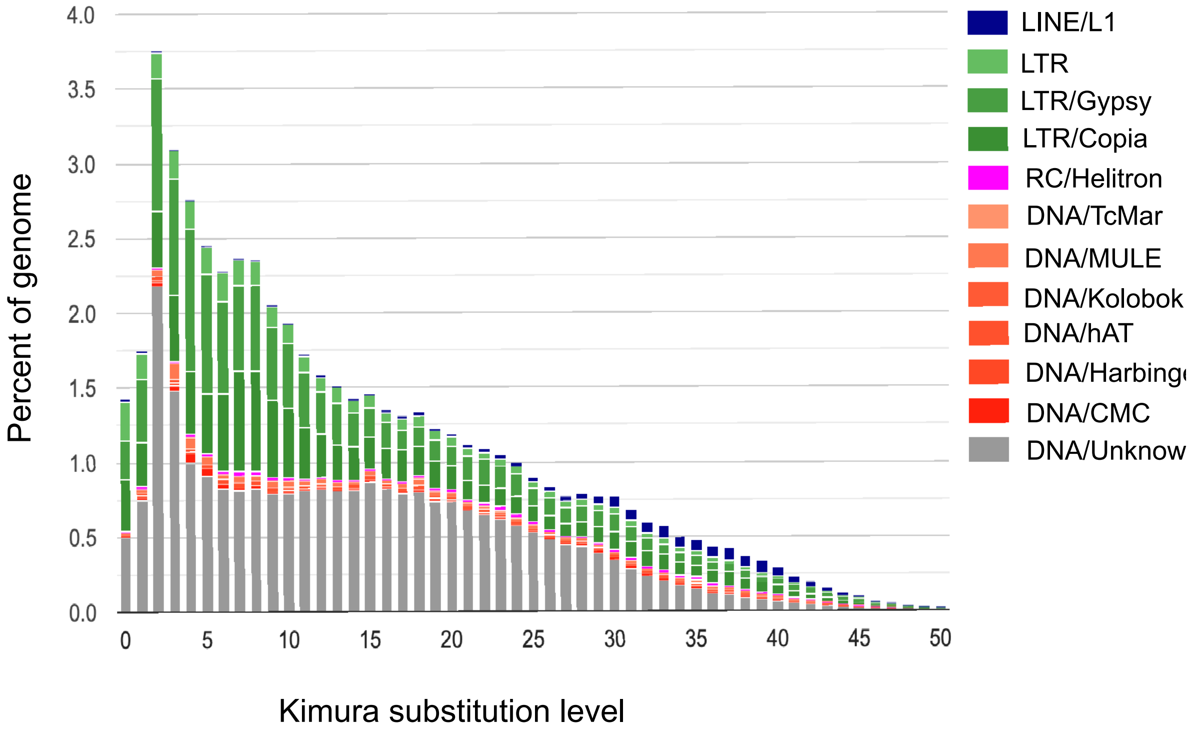

Supplemental Fig. S22. Interspersed repeat landscape. The interspersed repeat landscape, revealing the distribution and divergence of repeat elements across the genome. The percentages of the genome composed of various repeat elements (Y-axis) are categorized according to their Kimura substitution level (X-axis; CpG adjusted values ranging from 0 to 50), each bar in the plot represents a 1% sequence divergence bin, adjusted for CpG Kimura divergence. The landscape is represented by a stacked bar plot, where each color corresponds to a specific class of repeats. The plot illustrates the copy-divergence analysis of these elements, with older copies indicated by higher Kimura values towards the right, and more recent copies towards the left, indicating the evolutionary dynamics of the repetitive elements within the genome.

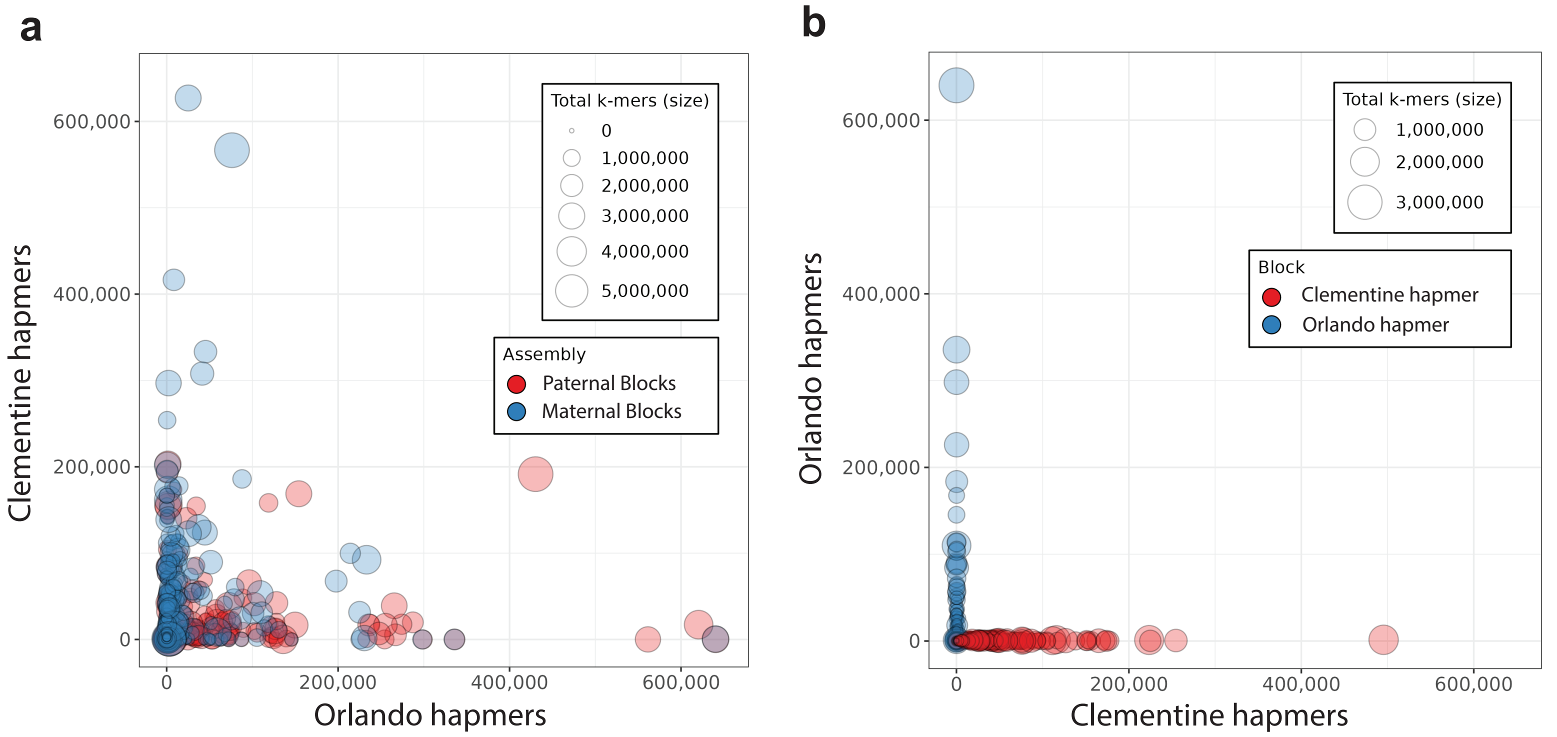

Fig. S23: Local haplotype phasing and ancestry assignment of ‘Fairchild’ genome assembly. (a) Proportion of parental k-mers identified in each phase block. Phase blocks with limited phase switching lie along the X-axis (for paternal) and Y-axis (for maternal). Off-axis points represent phase blocks with assignment of both maternal and paternal k-mers and are a sign of phase switching. (b) Proportion of parental k-mers identified in 100Kb windows across each phase block. Phase blocks with limited phase switching lie along the X-axis (for maternal) and Y-axis (for paternal).

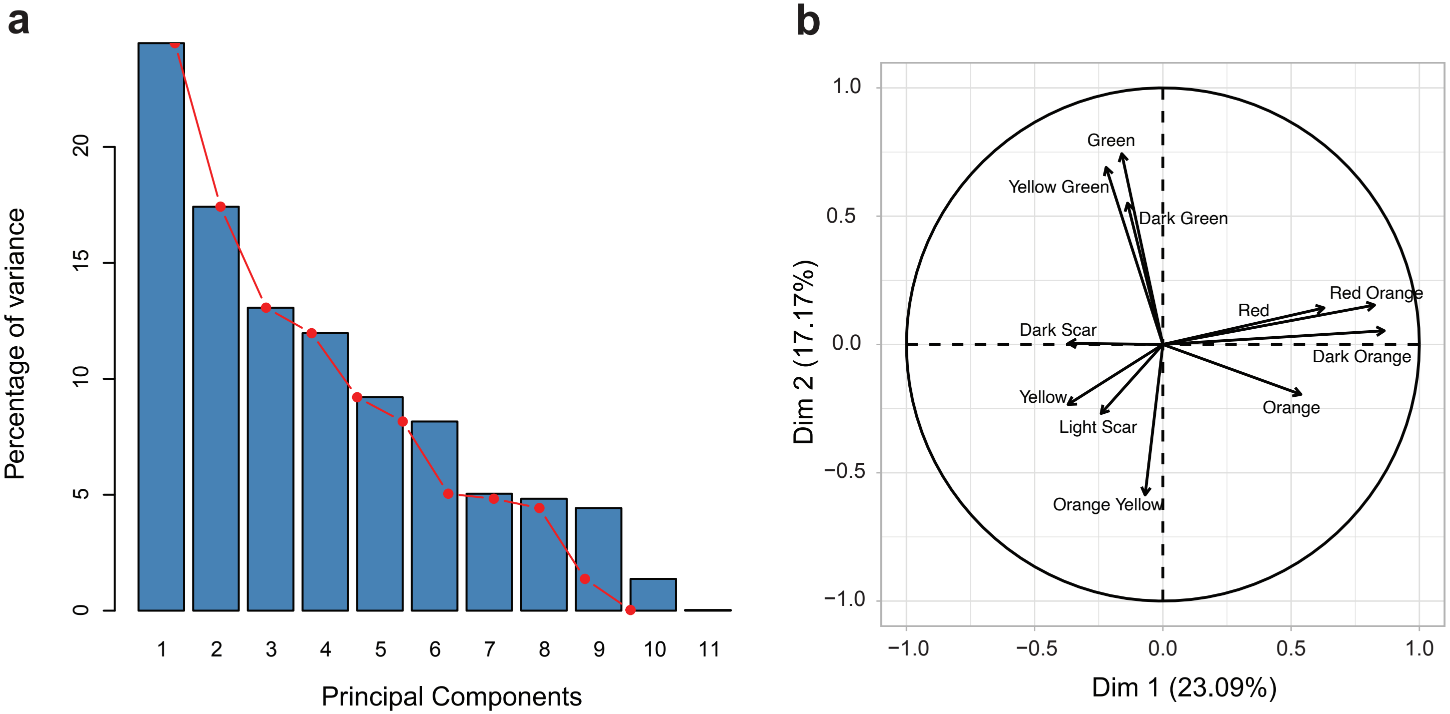

Fig. S24: Principal component analysis of fruit color using measurements for eleven colors. (a) Scree plot depicting percentage of variance in fruit color represented by each principal component. (b) Contribution of each color to the first two principal components, which were used as phenotypes in genome-wide association. The length of each arrow represents the magnitude of its contribution with respect to its direction along the first two principal components.
